# Preterm infants harbour diverse *Klebsiella* populations, including atypical species that encode and produce an array of antimicrobial resistance- and virulence-associated factors

**DOI:** 10.1099/mgen.0.000377

**Published:** 2020-05-21

**Authors:** Yuhao Chen, Thomas C. Brook, Cho Zin Soe, Ian O'Neill, Cristina Alcon-Giner, Onnicha Leelastwattanagul, Sarah Phillips, Shabhonam Caim, Paul Clarke, Lindsay J. Hall, Lesley Hoyles

**Affiliations:** ^1^​ Department of Surgery and Cancer, Faculty of Medicine, Imperial College London, London, UK; ^2^​ Department of Biomedical Sciences, Faculty of Science and Technology, University of Westminster, London, UK; ^3^​ Gut Microbes and Health, Quadram Institute Bioscience, Norwich Research Park, Norwich, UK; ^4^​ Bioinformatics and Systems Biology Program, School of Bioresources and Technology, King Mongkut's University of Technology Thonburi (Bang Khun Thian Campus), Bangkok, Thailand; ^5^​ Neonatal Intensive Care Unit, Norfolk and Norwich University Hospitals NHS Foundation Trust, Norwich, UK; ^6^​ Norwich Medical School, University of East Anglia, Norwich, UK; ^7^​ Department of Biosciences, School of Science and Technology, Nottingham Trent University, Nottingham, UK

**Keywords:** *Klebsiella oxytoca*, microbiome, shotgun metagenomics, taxonomy

## Abstract

*
Klebsiella* spp. are frequently enriched in the gut microbiota of preterm neonates, and overgrowth is associated with necrotizing enterocolitis (NEC), nosocomial infections and late-onset sepsis. Little is known about the genomic and phenotypic characteristics of preterm-associated *Klebsiella *, as previous studies have focused on the recovery of antimicrobial-resistant isolates or culture-independent molecular analyses. The aim of this study was to better characterize preterm-associated *Klebsiella* populations using phenotypic and genotypic approaches. Faecal samples from a UK cohort of healthy and sick preterm neonates (*n*=109) were screened on MacConkey agar to isolate lactose-positive *Enterobacteriaceae *. Whole-genome sequences were generated for *Klebsiella* spp., and virulence and antimicrobial resistance genes identified. Antibiotic susceptibility profiling and *in vitro* macrophage and iron assays were undertaken for the *Klebsiella* strains. Metapangenome analyses with a manually curated genome dataset were undertaken to examine the diversity of *Klebsiella oxytoca* and related bacteria in a publicly available shotgun metagenome dataset. Approximately one-tenth of faecal samples harboured *Klebsiella* spp. (* Klebsiella pneumoniae *, 7.3 %; *Klebsiella quasipneumoniae *, 0.9 %; *Klebsiella grimontii *, 2.8 %; *Klebsiella michiganensis *, 1.8 %). Isolates recovered from NEC- and sepsis-affected infants and those showing no signs of clinical infection (i.e. ‘healthy’) encoded multiple β-lactamases. No difference was observed between isolates recovered from healthy and sick infants with respect to *in vitro* siderophore production (all encoded enterobactin in their genomes). All *K. pneumoniae *, *K. quasipneumoniae *, *K. grimontii* and *K. michiganensis* faecal isolates tested were able to reside and persist in macrophages, indicating their immune evasion abilities. Metapangenome analyses of published metagenomic data confirmed our findings regarding the presence of *K. michiganensis* in the preterm gut. There is little difference in the phenotypic and genomic characteristics of *Klebsiella* isolates recovered from healthy and sick infants. Identification of β-lactamases in all isolates may prove problematic when defining treatment regimens for NEC or sepsis, and suggests that healthy preterm infants contribute to the resistome. Refined analyses with curated sequence databases are required when studying closely related species present in metagenomic data.

## Data Summary

16S rRNA gene sequence data associated with this article have been deposited at DDBJ/ENA/GenBank under BioProject accession PRJEB34372. The Whole Genome Shotgun project has been deposited at DDBJ/ENA/GenBank under BioProject accession PRJNA471164. The metagenome-assembled genomes are available from figshare. The published sequence data of Ward *et al.* [1] used to generate the metagenome-assembled genomes are available under BioProject accession number 63661.

**Impact Statement**

Polyphasic characterization of isolates recovered from the faeces of preterm infants has demonstrated that *Klebsiella* spp. recovered from these patients are genomically more diverse than previously recognized. All *Klebsiella pneumoniae *, *Klebsiella quasipneumoniae *, *Klebsiella grimontii* and *Klebsiella michiganensis* faecal isolates studied were able to reside and persist in macrophages, indicating their immune evasion abilities and potential for causing infections in at-risk infants. The identification of *K. michiganensis* in samples, and the abundance of *K. michiganensis* genomes in public repositories, adds to the growing body of evidence indicating that *K. michiganensis* is likely to be more clinically relevant than *Klebsiella oxytoca* in human-associated infections. Metapangenome analyses of publicly available shotgun metagenomic data confirmed the prevalence of *K. michiganensis* in the faeces of preterm infants, and highlighted the need for refined taxonomic analyses when splitting closely related species from one another in metagenomic studies.

## Introduction

The gut microbiota encompasses bacteria, archaea, lower eukaryotes and viruses, with these microbes contributing to host gastrointestinal (GI) and systemic health. Host–microbiome interactions within the intestine are particularly important in neonates, contributing to the development of the immune response, the establishment of the gut microbiome and protection from infections [2, 3]. Term infants (i.e. gestation 37 weeks) are rapidly colonized after exposure to the mother’s microbiota and the environment, with streptococci and *Enterobacteriaceae* dominating in the initial phases [2], and *Bifidobacterium* spp. becoming prominent as the infant grows [2].

In contrast, colonization of preterm infants (i.e.<37 weeks’ gestation) occurs in neonatal intensive care units (NICUs) and is shaped by the significant number of antibiotics [‘covering’ (i.e. to cover possible early onset infection from birth) and treatment] these infants receive in the first days and weeks post-birth. The microbiota in preterm infants is enriched for bacteria such as *Enterobacteriaceae *, *Enterococcus* and *Staphylococcus* [4, 5].

Critically, colonization of these at-risk infants with potentially pathogenic taxa, in concert with an unstable microbiome, and immaturity of their GI tract and immune system, is thought to contribute to nosocomial infections such as late-onset sepsis (LOS) or necrotizing enterocolitis (NEC) [6–12].

The family *Enterobacteriaceae* comprises more than 25 genera of catalase-positive, oxidase-negative Gram-negative bacteria and encompasses many pathogens (e.g. *Escherichia coli *, *Klebsiella pneumoniae *, *Shigella dysenteriae *, *Enterobacter cloacae *, *Serratia marcescens* and *Citrobacter* spp.] [13]. While coagulase-negative staphylococci are the most common cause of LOS in preterm infants, *Enterobacteriaceae* that translocate from the preterm gut to the bloodstream also cause this condition [8, 9]. In addition, *Enterobacteriaceae* are associated with higher morbidity than the staphylococci, and blooms in *Proteobacteria* – thought to be linked to impaired mucosal barrier integrity – have been reported immediately prior to the diagnosis of LOS [8, 9, 14]. Predictions made from shotgun metagenomic data show replication rates of all bacteria – and especially the *Enterobacteriaceae* and *Klebsiella* – are significantly increased immediately prior to NEC diagnosis [15]. This altered gut microbiome influences intestinal homeostasis and contributes to NEC [16], in tandem with the immature preterm immune system contributing to intestinal pathology in response to blooms of *Proteobacteria.*

Associations between *Klebsiella *-related operational taxonomic units (OTUs) and the development of NEC have been noted, suggesting that members of this genus contribute to the aetiology of NEC in a subset of patients [17, 18]. Although Sim *et al.* [17] found that one of their two distinct groups of NEC infants had an overabundance of a *Klebsiella* OTU, these researchers failed to identify a single predominant species of *Klebsiella *, recovering representatives of several genera (* K. pneumoniae *, *Klebsiella oxytoca *, *Klebsiella aerogenes *, *E. cloacae *, *E. coli* and *S. marcescens *) from samples. *Klebsiella* spp. and their fimbriae-encoding genes were significantly enriched in faeces collected immediately prior to the onset of NEC in a US infant cohort. These fimbriae may contribute to the overexpression of TLR4 receptors observed in preterm infants [15]. Confirming the role of these bacteria in NEC will require the reproduction of certain aspects of the disease in model systems, using well-characterized bacteria recovered from preterm infants [14, 17].

To date, there is limited information on the genomic and phenotypic features of preterm-associated *Klebsiella* spp. Thus, to characterize these important opportunistic pathogens, and to build a collection of preterm-associated *Klebsiella* strains for use in future mechanistic studies relevant to preterm-infant health, we isolated and characterized (phenotypically and genomically) bacteria from a cohort of preterm neonates enrolled in a study at the Norfolk and Norwich University Hospital (NNUH), Norwich, UK. Recovered *Klebsiella* isolates were subject to additional phenotypic tests that complemented genomic data. In addition, for the increasingly important species *K. oxytoca *, in which our laboratories have a specific interest, metapangenome analyses were undertaken to better understand the prevalence and potential virulence of this organism and related species in the context of the preterm neonate gut microbiota.

## Methods

### Collection of faecal samples

Faeces were collected from premature neonates (<37 weeks’ gestation) (Table S1, available in the online version of this article). The Ethics Committee of the Faculty of Medical and Health Sciences of the University of East Anglia (Norwich, UK) approved this study. The protocol for faeces collection was laid out by the Norwich Research Park (NRP) Biorepository (Norwich, UK) and was in accordance with the terms of the Human Tissue Act 2004 (HTA), and approved with licence number 11 208 by the Human Tissue Authority. Infants admitted to the NICU of the NNUH were recruited by doctors or nurses with informed and written consent obtained from parents. Collection of faecal samples was carried out by clinical researchers and/or research nurses, with samples stored at −80 °C prior to DNA extraction.

### 16S rRNA gene sequencing and analyses

DNA was extracted from samples using the FastDNA SPIN Kit for Soil (MP Biomedicals) and processed for sequencing and analyses as described previously [19]. This 16S rRNA gene sequence data associated with this project have been deposited at DDBJ/ENA/GenBank under BioProject accession PRJEB34372.

### Isolation of bacteria and biochemical characterization

For isolation work, a single faecal sample from each baby (*n*=109; Table S1) was thawed and 0.1 g homogenized in 1 ml TBT buffer (100 mM Tris/HCl, pH 8.0; 100 mM NaCl; 10 mM MgCl_2_·6H_2_O). Homogenates were serially diluted 10^−1^ to 10^−4^ in TBT buffer. Aliquots (50 µl) of homogenate were spread on MacConkey agar no. 3 (Oxoid Ltd) plates in triplicate and incubated aerobically at 37 °C overnight.

Differential counts (based on colony morphology) of all lactose-positive (i.e. pink) colonies were made in triplicate to calculate colony-forming units (c.f.u.) g^−1^ (wet weight) faeces. One of each colony type per plate was selected and restreaked on MacConkey agar three times for purification, with aerobic incubation at 37 °C overnight each time. A single colony from each pure culture was resuspended in 5 ml of sterile distilled water; the API 20E kit (bioMérieux) was used according to the manufacturer’s instructions to give preliminary identities for each of the isolates recovered.

### DNA extraction, whole-genome sequencing and assembly

DNA was extracted using a phenol–chloroform method fully described previously [20] from overnight cultures of strains, and sequenced using the 96-plex Illumina HiSeq 2500 platform to generate 125 bp paired-end reads [21]. Raw data provided by the sequencing centre were checked using fastqc v0.11.4 (https://www.bioinformatics.babraham.ac.uk/projects/fastqc/); no adapter trimming was required, and reads had an average Phred score >25. MetaPhlAn2.6 [22] was used to identify which species genome sequences represented. According to the results given by MetaPhlAn2.6, appropriate reference genomes were retrieved from Ensembl Genome (http://bacteria.ensembl.org/index.html) to guide reference-based assembly using BugBuilder v1.0.3b1 (default settings for Illumina data) [23]. Summary statistics for the *Klebsiella* genome sequences generated in this study, including accession numbers, can be found in Table S2. This Whole Genome Shotgun project has been deposited at DDBJ/ENA/GenBank under BioProject accession PRJNA471164.

### Genome analyses

Average nucleotide identity (ANI) between genome sequences of isolates and reference strains (* Klebsiella grimontii* 06D021^T^, GCA_900200035; *K. oxytoca* 2880STDY5682490, GCA_900083895; *Klebsiella michiganensis* DSM 25444^T^, GCA_002925905) was determined using FastANI (default settings) [24].

*K. oxytoca *, *K. michiganensis* and *K. grimontii* genomes were uploaded to the *Klebsiella oxytoca */*michiganensis* multilocus sequence typing (MLST) website (https://pubmlst.org/koxytoca/) sited at the University of Oxford [25] on 28 July 2019 to determine allele number against previously defined house-keeping genes (*rpoB*, *gapA*, *mdh*, *pgi*, *phoE*, *infB* and *tonB*). *K. pneumoniae* genomes were analysed using the Institut Pasteur MLST database (https://bigsdb.pasteur.fr/klebsiella/klebsiella.html). Kleborate [26, 27] and Kaptive [28] were used to identify capsular type and O antigen type.

Virulence genes were identified by blastp of genome amino acid sequences against the Virulence Factors of Pathogenic Bacteria Database (VFDB; ‘core dataset’ downloaded 27 July 2019) [29]; results are reported for >70 % identity and 90 % query coverage. Antimicrobial resistance (AMR) genes were identified by blastBLASTp against the Comprehensive Antibiotic Resistance Database (CARD) download (27 July 2019; protein homologue dataset) [30]; only strict and perfect matches with respect to CARD database coverage and bit-score cut-off recommendations are reported.

Genomic traits were visualized using anvi’o-5.5 [31] according to the pangenomic workflow. Briefly, for each figure presented herein, genomes were used to create an anvi’o contigs database, which contained open reading frames (ORFs) predicted using Prodigal v2.6.3 [32]. A multiple-sequence alignment was created using blastBLASTp. A Markov CL algorithm [33] was used to identify gene clusters (--mcl-inflation 10; high sensitivity for identifying gene clusters of closely related species or strain level). Gene clusters and genomes were organized using Euclidean distance and Ward linkage, with results visualized using Google Chrome.

### Phenotypic characterization of *Klebsiella* isolates

#### Iron assays

Pre-cultures of *Klebsiella* isolates (5 ml) were grown overnight in LB broth (37 °C, 160 r.p.m.). Aliquots (500 µl) were harvested (4000 r.p.m., 20 min) and the cell pellets washed twice with PBS. The cell suspensions (50 µl) were used to inoculate 5 ml cultures containing M9 minimal medium (Na_2_HPO_4_, 6.9 g l^−1^; KH_2_PO_4_, 3 g l^−1^; NaCl, 0.5 g l^−1^; NH_4_Cl, 1 g l^−1^; CaCl_2_, 0.1 mM; MgSO_4_, 2 mM; 0.2 % glucose) at 37 °C. At 20 h, bacterial growth and siderophore production were measured using the CAS assay [34]. An aliquot (100 µl) of the cell culture supernatant was mixed with CAS dye (100 µl), followed by the shuttle solution (4 µl) and siderophore production was monitored at 620 nm at 4 h using a BioRad Benchmark Plus microplate spectrophotometer. A decrease in the blue colour of the CAS dye was measured using uninoculated medium as control. The estimated amount of total siderophore produced by *Klebsiella* isolates was calculated using the CAS standard curve based upon a desferrioxamine B standard (1 : 1).

#### Macrophage assays

All strains were grown on LB broth+1.5 % agar and incubated overnight at 37 °C. THP-1 monocytes were obtained from ATCC (TIB-202) and were maintained in RPMI (Gibco: 72400021) plus 10 % heat-inactivated foetal bovine serum (FBS; Gibco: 10500064) in a humidified incubator at 37 °C with 5 % CO_2_. THP-1 monocytes were differentiated into macrophages in RPMI+10 mM HEPES+10 % FBS+10 ng ml^−1^ phorbol 12-myristate 13-acetate (PMA; Sigma) and seeded at 1×10^5^ cells per well in a 96-well tissue culture dish and incubated for 15 h. Overnight cultures of bacteria were diluted 1 : 100 into fresh LB broth and grown until mid-exponential phase. Bacteria were then washed twice with PBS and diluted to 1×10^7^ c.f.u. ml^−1^ in RPMI+10 mM HEPES+10 % FBS and 100 µL of bacteria was added to each well. Plates were then centrifuged at 300 ***g*** for 5 min to synchronize infections. Bacteria–macrophage co-culture was incubated at 37 °C/5 % CO_2_ for 30 min to allow for phagocytosis. Cells were then washed three times in PBS and medium was replaced with above culture medium supplemented with 300 µg ml^−1^ gentamicin and 100 units ml^−1^ polymyxin B to eliminate extracellular bacteria. Cell were again incubated at 37 °C/5 % CO_2_ for 1.5 h. Cells were then washed three times with PBS, with medium for cells at later time points being replaced with culture medium supplemented with 300 µg ml^−1^ gentamicin, and incubated for a further 4.5 h. Intracellular bacterial load was enumerated by lysing macrophages in PBS+1 % Triton X-100 for 10 min at room temperature, serially diluting cultures and plating on LB agar. Plates were incubated overnight at 37 °C and colonies were counted the following day.

#### Calculation of antibiotic minimal inhibitory concentration (MIC) for the *Klebsiella* isolates

The broth microdilution method was used to calculate the MIC of the *Klebsiella* isolates. Serial twofold dilutions of benzylpenicillin, gentamicin and meropenem were added to sterile nutrient broth. The antibiotics used in this assay were supplied by the NICU of NNUH. The inoculum for each of the isolates was prepared using 10 ml from a fresh overnight culture. Microplates were incubated for 24 h at 37 °C under aerobic conditions. Optical density was monitored using a plate reader (BMG Labtech, UK) at 595 nm. MICs were determined as the lowest concentration of antibiotic inhibiting any bacterial growth. All experiments were repeated in triplicate. For the aminoglycoside gentamicin and the carbapenem meropenem, *Klebsiella* (* Enterobacteriaceae *) breakpoints were determined according to European Committee on Antimicrobial Susceptibility Testing (EUCAST) guidelines (version 8.1, published 16 May 2018, http://www.eucast.org/fileadmin/src/media/PDFs/EUCAST_files/Breakpoint_tables/v_8.1_Breakpoint_Tables.pdf). No EUCAST data were available for benzylpenicillin (EUCAST states this aminopenicillin has no clinically useful activity against *Enterobacteriaceae *).

### Estimation of abundance of *K. oxytoca* in shotgun metagenomic data

We chose to analyse a published preterm gut metagenome dataset [1] in this study, as it had been previously used to identify associations between uropathogenic *E. coli* and NEC. Trimmed, human-filtered, paired-end read data deposited in the Sequence Read Archive by Ward *et al.* [1] are available under BioProject accession number 63 661. Information on Ward *et al*.’s samples included in this study can be found in Table S3. Ward *et al.* [1] used MetaPhlAn to determine the abundance of bacteria in samples. However, the marker genes used to enumerate *K. oxytoca* in the MetaPhlAn2.6 database are derived from 11 genomes, 5 of which are not *K. oxytoca* (* Raoultella ornithinolytica* 10–5246, GCF_000247895; *K. michiganensis* E718, GCF_000276705; *K. michiganensis* Kleb_oxyt_10–5250_V1, GCF_000247915; *K. michiganensis* KCTC 1686, GCF_000240325; *K. michiganensis* Kleb_oxyt_10–5242_V1, GCF_000247835). Therefore, the relative abundance of bacteria was instead determined using Centrifuge [35]. While MetaPhlAn2.6 relies on a precompiled database of unique marker genes for determining taxonomic abundance, the Centrifuge database can be updated at will using genomes downloaded from the National Center for Biotechnology Information (NCBI) database. A bacteria- and archaea-specific complete genome database was generated for use with Centrifuge via the NCBI database on 1 July 2018. Species-level abundances, based on read-level data, for *K. oxytoca* and *K. michiganensis* in the study of Ward *et al.* [1] were determined. (NB: *K. grimontii* genomes were not included in the Centrifuge database, nor are they included in MetaPhlAn2.6 or the most-recent version of Kraken2.)

### Metapangenome analyses of *K. oxytoca *, *K. michiganensis* and *K. grimontii*

A total of 162 *K. oxytoca *-related whole-genome sequences were retrieved from GenBank on 31 May 2018 (Table S4). On the basis of *bla*_OXY_, phylogenetic and ANI analyses [36], these had been confirmed to belong to *K. oxytoca* (*n*=64), *K. grimontii* (*n*=24) and *K. michiganensis* (*n*=74) (Table S5). Prokka v1.13.3 [37] was used to annotate the 162 downloaded and 5 infant genomes. The resulting .gff files were subjected to pangenome analyses using Roary v3.12.0 (default settings) [38]. Genes present in 165–167 strains were defined as the core cluster, while those present in 25–164 strains were defined as the accessory cluster. The remaining genes that only existed in single strains were classified into strain-specific clusters. FastTree v2.1.10 [39] was used to generate a phylogenetic tree from the core gene alignment, with the tree visualized using FigTree v1.4.4 (http://tree.bio.ed.ac.uk/software/figtree/).

PanPhlAn (panphlan_pangenome_generation.py v1.2.3.6; panphlan_profile.py v1.2.2.3; panphlan_map.py v1.2.2.5 [40]) was used to profile strains within metagenomes using the Roary-generated pangenome dataset. Gene family clusters across all 167 available genomes and centroid sequence files outputted from Roary were uploaded to PanPhlAn to build a Bowtie2-indexed pangenome database, against which raw reads (concatenated read pair files) were mapped using Bowtie2 v2.3.0. The coverages of all gene positions were detected and extracted using samtools v1.4.1, and then integrated to a gene family coverage profile for each sample. Pangenome-related strains were predicted to exist if a consistent and similar coverage depth across a set of 5774 gene families was detected under the non-default parameters of PanPhlAn (--min_coverage 1, --left_max 1.70, --right_min 0.30; panphlan_profile.py). Principal component analysis (PCA) was performed on 500 accessory genes randomly selected from the pangenome using the R package FactoMineR [41], allowing us to distinguish different species at the gene level.

### Recovery of metagenome-assembled genomes (MAGs) from metagenomes

For metagenome samples in which *K. oxytoca *-related strains were identified using PanPhlAn, attempts were made to recover them as MAGs. All reads in samples were mapped against the pangenome database using Bowtie2 and mapped paired-end reads were extracted by using FastQ Screen v0.11.3 as new fastq files (with parameter --tag, --filter 3). The extracted paired-end reads were assembled using SPAdes v3.12.0 [42]. These assemblies were known as original MAGs. A genome size of 5.5 Mb was set as a strict threshold: any assembly whose genome size was lower than this threshold was not considered in downstream analyses. FastANI was applied to calculate the ANI (cut-off 95 %) between MAGs and the three species reference genomes to double-check the predominant species in corresponding samples. The quality of each MAG was assessed using CheckM v1.0.18 [43].

A small number of the original MAGs were of high quality [44], but some contained a large number of contaminant contigs. All original MAGs were decontaminated as follows. Coding sequences of species-specific genomes in the pangenome were predicted using Prodigal v2.6.3 [32] with default settings and the resulting multi-FASTA files containing protein sequences were concatenated to single files, which were used to build *K. oxytoca *-, *K. michiganensis *- and *K. grimontii *-specific databases in Diamond [45] format. Contigs of the original MAGs were aligned against the corresponding database under different minimum identity (%) cut-offs to report sequence alignments (with parameter --id 95, 96, 97, 98, 99 and 100). All unmapped contigs and contigs <500 nt in length were discarded from the original MAGs and the quality of the new MAGs acquired was evaluated again using CheckM, to identify a Diamond blast identity threshold at which decontamination was effective while maintaining high genome completeness.

## Results and discussion

### Composition of the microbiota of preterm neonates

**Fig. 1.**
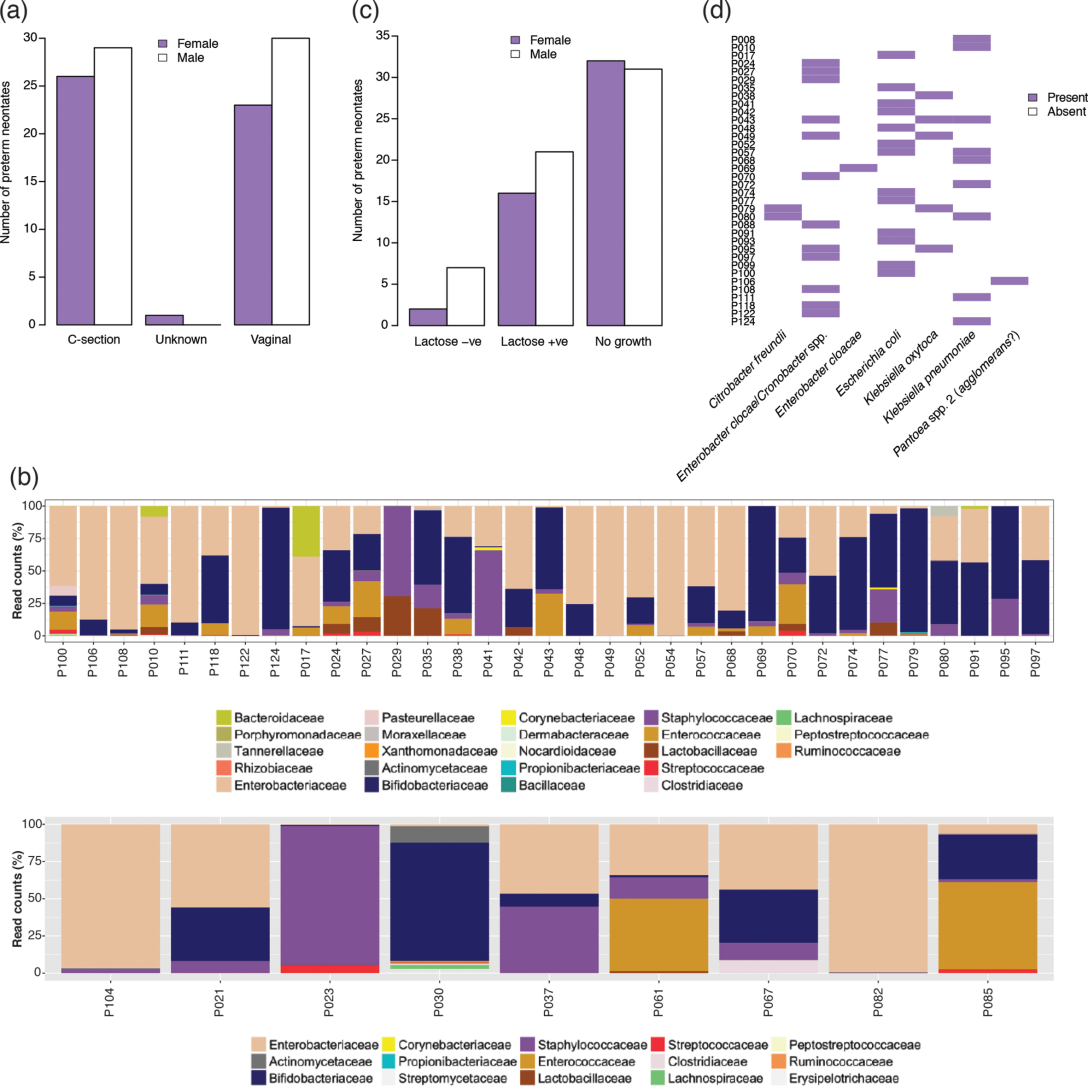
Summary information for UK cohort included in this study. (a) Breakdown of birth mode and sex of preterm neonates. (b) 16S rRNA gene sequence results for *Enterobacteriaceae *-positive samples: upper panel, samples from which lactose-positive isolates were recovered; lower panel, samples from which lactose-negative isolates were recovered. (c) Representation of lactose-negative and lactose-positive *Enterobacteriaceae* isolated from faecal samples. (d) Tentative identities of lactose-positive *Enterobacteriaceae* as determined by using API 20E.

The first faecal samples available after birth were collected from 109 hospitalized preterm infants (*n*=50 female; *n*=59 male) in the NICU of the NNUH (Table S1**,** Fig. 1a). On the basis of 16S rRNA gene sequencing data, *Enterobacteriaceae* were detected in the faeces of 42 (38.5 %) of the infants (Fig. 1b).

All faecal samples were screened for *Enterobacteriaceae* using MacConkey agar no. 3 (Fig. 1c). Forty-six (42.2 %) samples were positive for *Enterobacteriaceae* (*n*=9 lactose-negative, carriage rate 8.3 %; *n*=37 lactose-positive, carriage rate 33.9 %). Lactose-negative isolates were not characterized further. API 20E was used to provide tentative identification of lactose-positive *Enterobacteriaceae* (LPE) from 36 neonates (isolate could not be resuscitated for neonate P054) (Fig. 1d). Of the 36 infants from whose faeces isolates were recovered, 23 were healthy, 3 had or were subsequently diagnosed with NEC, 8 had suspected sepsis, 1 had an operation for gastroschisis and 1 was diagnosed with an eye infection after the faecal sample was taken (Table S1).

### Whole-genome sequencing of neonatal faecal LPE

Whole-genome sequences were obtained for 56 LPE. MetaPhlAn2.6 was used to assign identities to genomes (not shown). Among the 56 isolates sequenced, 20 were identified as *K. pneumoniae* (carriage rate 8.3 %; Table S2), 14 were *E. cloacae* complex (carriage rate 11.9 %), 13 were *E. coli* (carriage rate 11.9 %), 5 were *K. oxytoca* (Table S2), 2 were *Citrobacter freundii* (carriage rate 1.8 % [46]), 1 was *Citrobacter murliniae* (carriage rate 0.9 % [46]) and 1 was *R. ornithinolytica* (carriage rate 0.9 % [47]). *E. coli* and *E. cloacae* complex isolates will be discussed in detail elsewhere. MetaPhlAn2.6-generated identities matched those given by API 20E (Table S2).

Reference-based assembly of genomes was performed using BugBuilder [23] (Table S2). To determine whether preterm neonates may harbour more than one strain of a species in their faecal microbiota, nine isolates (#64–#73) were collected from neonate P008. These had all been identified as *K. pneumoniae* by API 20E and genome data. ANI across the nine isolates was >99.99 %. To determine whether the isolates were identical, gene content analysis was performed using Roary [38]. The average number of coding sequences (CDSs) among these isolates was 5385 (6.25) (Fig. S1a–d). Anvi’o showed that the isolates were highly similar (Fig. S1e). Isolates of the same species from other neonates were also found to be identical to one another (#102 and #103 from P080; #118 and #119 from P124). For sets of identical isolates, only one was taken forward for further analyses. This left 14 distinct *Klebsiella* strains (9 *K. pneumoniae *; 5 *K. oxytoca *) for further analyses.

### Genome analyses of *K. pneumoniae* strains

*K. pneumoniae* is a commensal of the human gut microbiota and can cause nosocomial infections, NEC and LOS in premature neonates [9, 17, 48–51]. The genetic backgrounds of the neonate isolates were explored to determine virulence and the AMR genes encoded within the strains’ genomes.

**Fig. 2.**
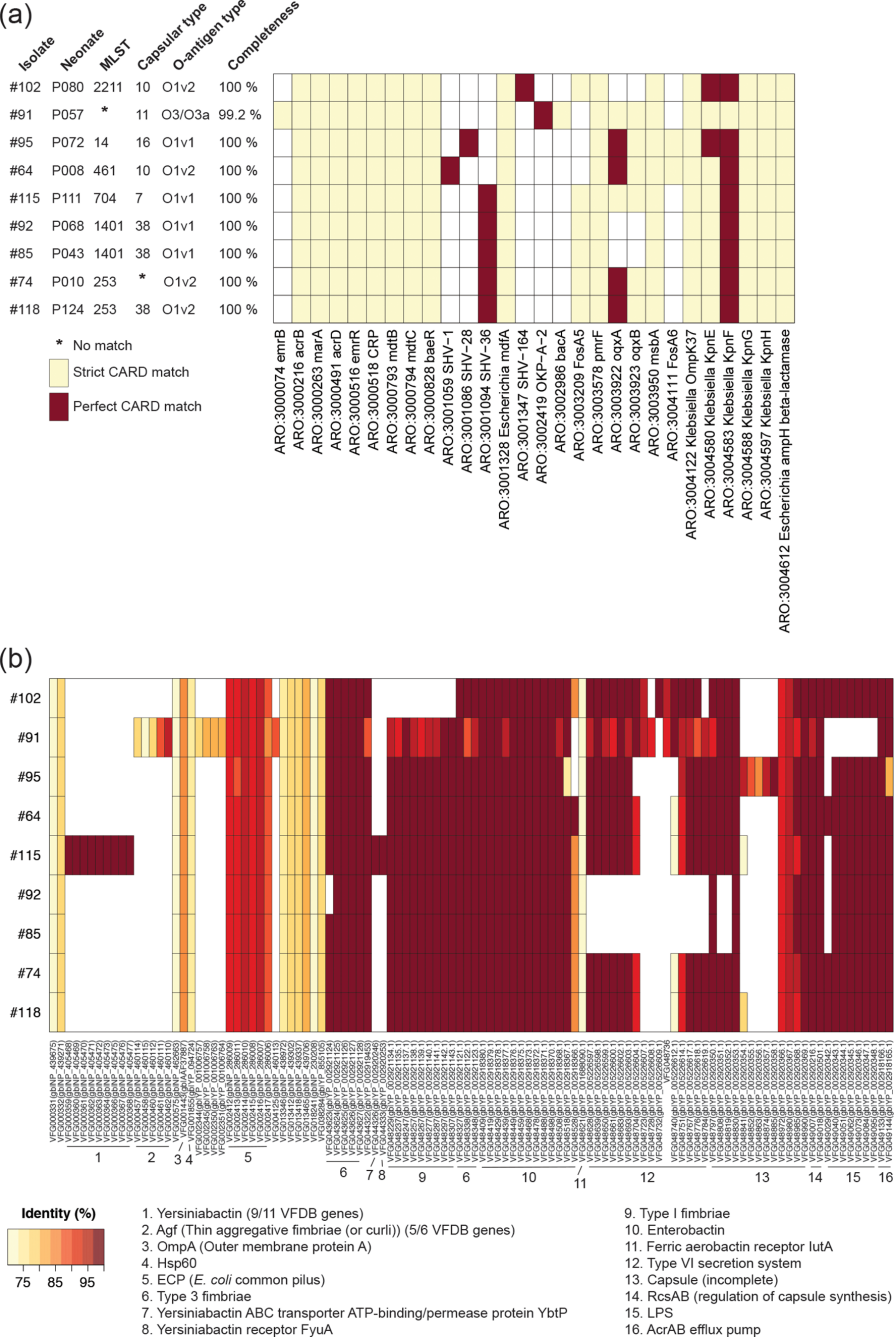
Summary of (a) antibiotic resistance and (b) virulence factor genes found in the *K. pneumoniae* isolates by comparison of protein sequences with those of the CARD and VFDB, respectively. (a) Strict CARD match, not identical but the bit score of the matched sequence is greater than the curated blastp bit score cut-off; perfect CARD match, 100 % identical to the reference sequence along its entire length. Loose matches are not shown to avoid presenting false positives based on sequences with low homology and bit scores below CARD blastp cut-off recommendations. (b) Identity (%), blastp only reported for those proteins sharing >70 % identity and 90 % query coverage with VFDB protein sequences.

Each isolate was genetically different, i.e. no two infants harboured the same strain of *K. pneumoniae* (Fig. S2). ANI analyses with representative strains of the seven phylogenetic groups of *K. pneumoniae* [52] showed that eight of the neonatal isolates were *K. pneumoniae* [98.83–98.98 % ANI with *K. pneumoniae* ATCC 13883^T^ (GCA_000742135)] and one (#91) was *K. quasipneumoniae* [98.5 % ANI with *K. quasipneumoniae* subsp. *quasipneumoniae* 01A030^T^ (GCA_000751755)]. MLST identified six sequence types (STs) within the *K. pneumoniae* strains (Fig. 2a). *K. quasipneumoniae* #91 had a novel *mdh* allele, so no ST could be specified for this strain. None of the STs belonged to clonal complex (CC) 258, responsible for hospital outbreaks due to its frequent carriage of *K. pneumoniae* carbapenemase (KPC) and other acquired AMR genes [53]. The capsule of *K. pneumoniae* and related species is considered one of its major virulence factors. K1, K2 and K5 capsular types and hypervirulent types have strong associations with human infectious diseases [54, 55]. None of our neonatal isolates had a capsular type commonly associated with infections or hypervirulent *K. pneumoniae *, although K7, K10, K11, K16 and K38 isolates have previously been recovered from clinical samples in Taiwan [56]. Although the capsular type of strain #74 was identified as K62 with 99.33 % confidence and 100 % coverage, there was one gene (*KL62-12*, according to Kaptive) missing from the locus, leaving it a non-perfect match. Further analyses showed the genes associated with K62 to be disrupted in strain #74 and not encoded on a contiguous stretch of DNA (Fig. S3). Of the nine *K. pneumoniae* strains analysed using Kleborate [26, 27], O1v1 and O1v2 were represented equally among the O-antigen types (*n*=4 for both). These can be distinguished using genomic data but are serologically cross-reactive [27]. *K. quasipneumoniae* #91 was O3/O3a.

The vast majority (e.g. ~90 % in the NNUH NICU) of preterm infants receive antibiotics during their NICU stay, often started routinely from admission (i.e. covering) if they are born very prematurely and/or have very low birth weight. Administration of antibiotics can lead to disruption of early colonization by microbes, potentially encouraging growth of opportunistic pathogens such as LPE, creating a selection pressure that may promote the development of AMR. All strains encoded homologues of *acrB*, *acrD*, *marA*, *emrR*, *CRP*, *mdtB*, *mdtC*, *baeR*, *Escherichia mdfA*, PmrF, *msbA*, OmpK37, KpnE, KpnF, KpnG, KpnH and *Escherichia ampH* β-lactamase, associated with antibiotic efflux and its regulation (*acrB*, *acrD*, *marA*, *emrR*, *CRP*, *mdtB*, *mdtC*, *baeR*) [57–59] and resistance to aminoglycosides; cationic antimicrobial peptides and antibiotics such as polymyxin (PmrF [60]); chloramphenicol (*mdfA* [61]), cefotaxime and cefoxitin (OmpK37); cefepime, ceftriaxone, colistin, erythromycin, rifampin, tetracycline and streptomycin [as well as enhanced sensitivity toward sodium dodecyl sulfate, deoxycholate, dyes, benzalkonium chloride, chlorohexidine and triclosan (KpnE, KpnF [62])]; azithromycin, ceftazidime, ciprofloxacin, ertapenem, erythromycin, gentamicin, imipenem, ticarcillin, norfloxacin, polymyxin-B, piperacillin, spectinomycin, tobramycin and streptomycin (KpnG, KpnH [63]); β-lactams and penicillin (*Escherichia ampH* β-lactamase [64]). As expected, the core genes *bla*_SHV_ and *bla*_OKP_, respectively, were found in *K. pneumoniae* and *K. quasipneumoniae* genomes [53]. *K. quasipneumoniae* #91 also encoded homologues of the acquired AMR gene *emrB* (a translocase that recognizes substrates, including carbonyl cyanide *m*-chlorophenylhydrazone, nalidixic acid and thioloactomycin) and *bacA* (confers resistance to bacitracin) (Fig. 2a). Plasmid-encoded *bla*_SHV_ enzymes represent an important subgroup of class A β-lactamases, while chromosomally encoded β-lactamase *bla*_OKP_ cannot hydrolyze extended-spectrum cephalosporins [65]. Homologues of the core AMR genes *oqxA* and *oqxB* (encoding OqxAB, a plasmid-encoded efflux pump that confers resistance to fluoroquinolones) were encoded by #64, #74, #91, #95, #115 and #118. Strains #64 and #95 encoded homologues of FosA6, while #74, #85, #92, #115 and #118 encoded homologues of FosA5 (both gene products confer resistance to fosfomycin and are core AMR genes [53]).

While the majority of the neonatal *K. pneumoniae* strains did not represent known pathogenic lineages, virulence factors were detected in their genomes using VFDB (Fig. 2b). The host limits iron availability within the GI tract to prevent colonization by pathogens and bacterial overgrowth. However, *Klebsiella* spp. have evolved numerous mechanisms to circumvent these defences. Thus, we determined whether gene clusters associated with iron uptake and siderophore systems (i.e. enterobactin, yersiniabactin, aerobactin, colibactin, salmochelin) were present in the strains. All strains encoded enterobactin, while only #115 encoded an additional system (yersiniabactin) (Fig. 2b). All strains encoded *E. coli* common pilus, OmpA, Hsp60, type 3 fimbriae, ferric aerobactin receptor *IutA* and the AcrAB efflux pump. All strains except #102 encoded type 1 fimbriae; all strains except *K. quasipneumoniae* #91 encoded typical *K. pneumoniae* lipopolysaccharide (LPS) according to VFDB (>70 % amino acid identity and 90 % query coverage). *K. quasipneumoniae* #91 encoded thin aggregative fimbriae, associated with biofilm formation and adhering to human mucosal or epithelial surfaces. Incomplete coverage of *Klebsiella* capsule genes is likely due to the limited database of VFDB compared with those used to populate Kaptive and Kleborate. Kaptive had shown #91 to be K11 and O3/O3a. The core LPS region of #91 was identified using the *waa* gene cluster [66]; WaaL clustering with an 80 % threshold showed that the strain had LPS core type 1 (Fig. S4) [67].

### Whole-genome analyses of isolates tentatively identified as *K. oxytoca*

*K. oxytoca* is a minor member of the human gut microbiota, recovered at low levels from the faeces of 1.6–9 % of healthy adults [68]. Toxigenic *K. oxytoca* is a causative agent of antibiotic-associated haemorrhagic colitis, a condition affecting mainly young and otherwise healthy outpatients after brief treatment with penicillin derivatives [69]. *K. oxytoca* has been detected in the faeces of a subset of preterm infants via cultivation or shotgun metagenomics, but its association with preterm-associated infections is unknown [1, 17, 70]. At the DNA level, bacteria characterized phenotypically as *K. oxytoca* actually represent six phylogroups/distinct species: Ko1, *K. michiganensis *; Ko2, *K. oxytoca *; Ko3, *Klebsiella spallanzanii *; Ko4, *Klebsiella pasteurii *; Ko6, *K. grimontii *; Ko8, *K. huaxiensis* [71–75]. *K. michiganensis* and *K. oxytoca* are distinguishable based on the *bla*_OXY_ gene they carry (*bla*_OXY-1_ and *bla*_OXY-2_, respectively) [71]. *K. grimontii* was recently described to accommodate Ko6 strains based on *rpoB*, *gyrA* and *rrs* gene sequences [73]. All six members of the complex can be differentiated by matrix-assisted laserdesorption ionization/time-of-flight mass spectrometry (MALDI-TOF MS) [75], but reference databases currently in routine clinical use lack reference spectra for the different species to allow identification beyond *K. oxytoca *. Consequently, reports on complex members other than *K. oxytoca* have only recently begun to appear in the literature [73–77]. The colonization of humans with *K. oxytoca* phylogroups has previously been associated with the genetic backgrounds of strains: Ko2 mainly inhabits the lower GI tract, with Ko1 and Ko6 generally associated with respiratory isolates and faecal isolates, respectively [72].

**Fig. 3.**
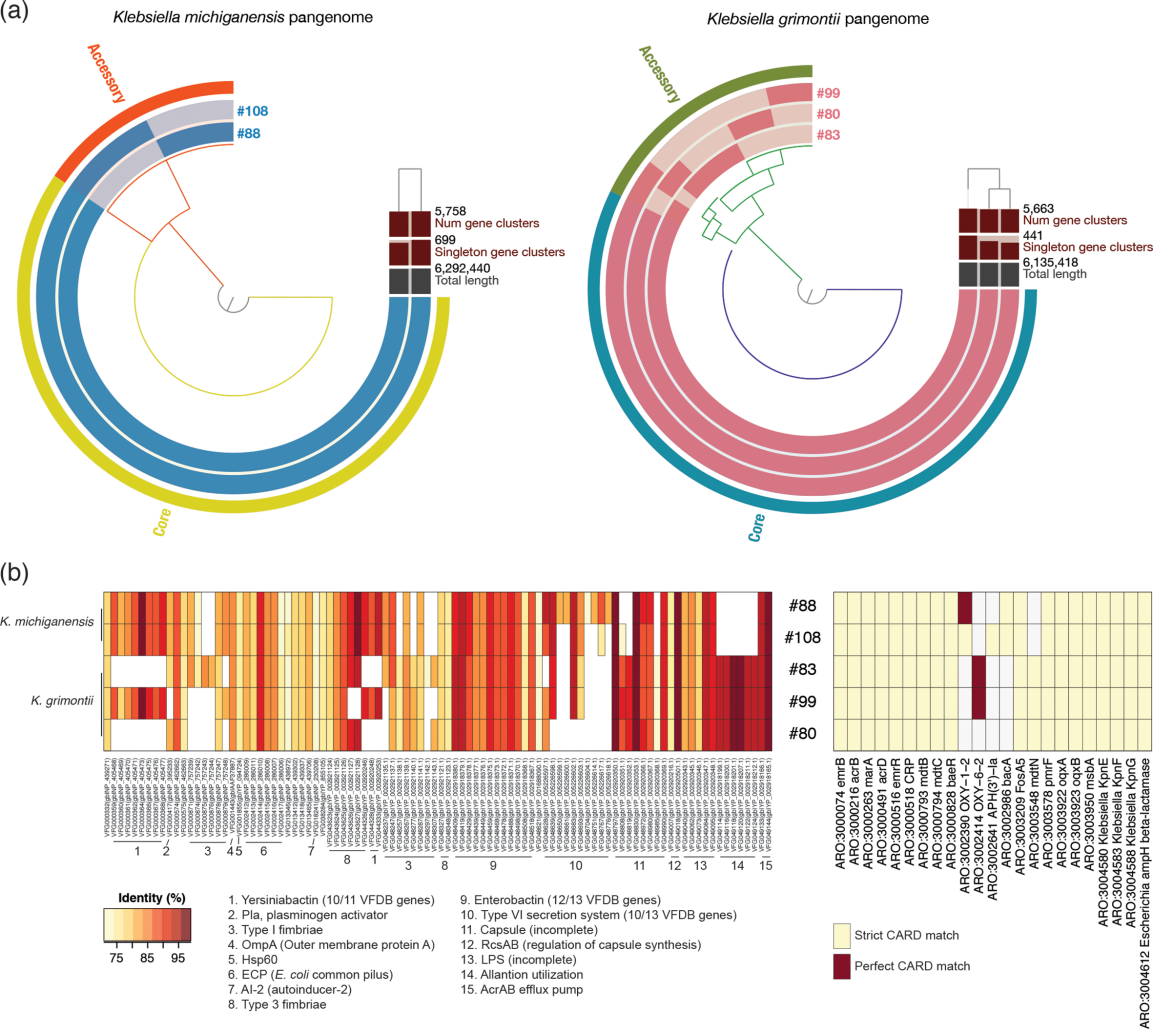
Genomic characterization of the *K. michiganensis* and *K. grimontii* isolates recovered from neonates. (a) Anvi’o representation of the genomes of *K. michiganensis* and *K. grimontii* isolates recovered from different infants. It is clear the isolates are different from one another at the genomic level. (b) Virulence factor (left side) and antibiotic resistance (right side) genes encoded by the isolates. Criteria for identity and strict/perfect match with respect to VFDB and CARD, respectively, are the same as those given for Fig. 2.

On the basis of API 20E data and initial genome (MetaPhlAn2.6) analysis, 5 neonatal isolates were identified as *K. oxytoca* (#80, #83, #88, #99, #108). It has recently been shown that API 20E and MALDI-TOF MS using current clinical reference databases are as effective as one another for the characterization of complex members as *K. oxytoca* [78]. MetaPhlAn2.6 cannot distinguish among species of the *K. oxytoca* complex. ANI of the genomes against reference genomes showed #88 and #108 to be *K. michiganensis* (both 98.78 and 98.94 % ANI, respectively, with GCA_002925905) and #80, #83 and #99 to be *K. grimontii* (99.18, 99.23 and 99.17 % ANI, respectively, with GCA_900200035) (Fig. S5a), with ANI cut-off values well above the ~95 % proposed for species delineation [79–81] and used by Passet and Brisse [73] to separate *K. grimontii* from *K. oxytoca* and *K. michiganensis *. Phylogenetic analysis with a panel of authentic *K. oxytoca *, *K. grimontii* and *K. michiganensis* genomes confirmed the species affiliations of the infant isolates (Fig. S5b). Similar to the *K. pneumoniae* isolates, no two infants harboured the same strain of *K. michiganensis* or *K. grimontii* (Fig. 3a).

It is notable that of the publicly available genomes deposited as *K. oxytoca *, 74 were found to represent *K. michiganensis *, 64 were *K. oxytoca* and 24 were *K. grimontii *. This suggests that *K. michiganensis* may be more clinically relevant than *K. oxytoca sensu stricto. K. michiganensis* was originally proposed to describe an isolate closely related to *K. oxytoca* recovered from a toothbrush holder [74]. The bacterium is now recognized as an emerging pathogen, with this recognition due to improved genomic characterization of clinical isolates that would have previously been described as *K. oxytoca* based on simple phenotypic tests or MALDI-TOF MS [78, 82–84].

### Predicted virulence and AMR determinant genes of infant-associated *K. michiganensis* and *K. grimontii*

The *K. michiganensis* and *K. grimontii* strains were examined for the presence of virulence-associated loci found in *K. pneumoniae* strains [53] (Fig. 3b). Enterobactin was encoded by all strains. Yersiniabactin was predicted to be encoded by *K. grimontii* #99 and *K. michiganensis* #88 and #108. Other siderophore-associated gene clusters (aerobactin, colibactin and salmochelin) found in *K. pneumoniae* were absent. An allantoinase gene cluster (including *allB*/*C*/*R*/*A*/*S* and *ybbW*), which plays a role in *K. pneumoniae* liver infection [85], was identified in the three *K. grimontii* strains.

Due to the clinical importance of AMR in *Enterobacteriaceae *, an *in silico* AMR gene profile was established for the *K. michiganensis* and *K. grimontii* strains. Homologues of 18 AMR determinant genes (acquired AMR genes – *emrB*, *emrR*; core AMR genes *acrB*, *acrD*, CRP, *marA*, *mdtB*, *mdtC*, *baeR*, FosA5, *pmrF*, *oqxA*, *oqxB*, *msbA*, KpnE, KpnF, KpnG, *Escherichia ampH* β-lactamase) were common to the 5 strains, similar to the *K. pneumoniae* isolates. Both *K. michiganensis* strains encoded homologues of OXY-1-2 [β-lactamase specific to *K. michiganensis* (Ko1 [86]) and *bacA*, while #108 encoded a homologue of *aph(3′)-la* (aminoglycoside phosphotransferase]. All *K. grimontii* strains encoded *mdtN* (potentially involved in resistance to puromycin, acriflavine and tetraphenylarsonium chloride), while #83 and #99 encoded homologues of OXY-6-2 (β-lactamase specific to *K. grimontii* (Ko6 [86]).

### Phenotypic characterization of *K. pneumoniae *, *K. quasipneumoniae *, *K. michiganensis* and *K. grimontii* neonatal isolates

Five of the 13 *Klebsiella* strains we characterized were isolated from preterm infants who had been diagnosed with either NEC or sepsis. Thus, we sought to link our genotypic analyses with clinically important virulence traits, including the ability to survive and replicate in host immune cells (i.e. macrophages) and the ability to produce iron-acquiring siderophores. We also determined the strains’ AMR profiles for a limited set of antimicrobials.

**Fig. 4.**
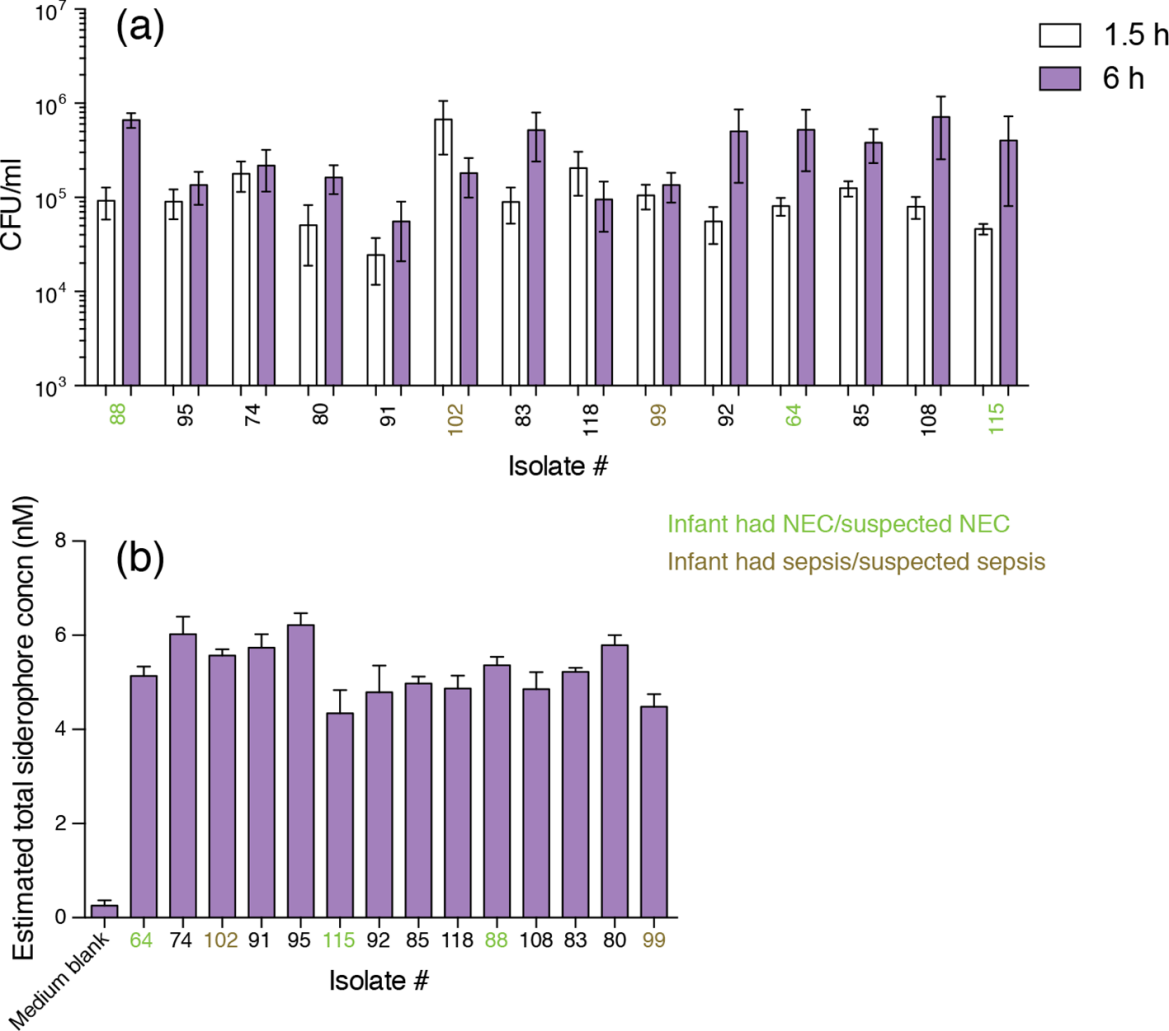
Phenotypic assays for the *Klebsiella* isolates recovered from infants. (a) Strains were tested for persistence in PMA-differentiated THP-1 macrophages using a gentamicin protection assay. Intracellular bacteria were enumerated 1.5 and 6 h after infection to determine persistence (*n*=4). Results are shown as mean (sd). (b) *Klebsiella* strains were grown in minimal medium and at 20 h bacterial growth (OD_600_) siderophore production was measured using the CAS assay (*n*=3). Results are shown as mean (sd).

Previous studies have indicated that respiratory infection-associated *K. pneumoniae* are able to survive within macrophages, a critical innate immune cell type required for optimal pathogen clearance [87]. However, to date there is limited information relating to this ability in gut-associated strains, and there is no information on other *Klebsiella* species. Thus, all *Klebsiella* strains isolated in this study were tested in PMA-differentiated THP-1 macrophages using a gentamicin protection assay. All strains appeared to persist within macrophages, as bacterial load was either maintained over the time course or increased or decreased between 1.5 and 6 h, although these values were not statistically significant (Fig. 4a). These data suggest that all *Klebsiella* strains tested can reside and persist in macrophages. This ability of all strains to survive, and in some cases potentially replicate, within macrophages indicates their immune evasion capabilities, which may link to increased risk and incidence of NEC and sepsis if these strains translocate from the ‘leaky’ preterm GI tract to systemic sites, contributing to the inflammatory cascades characteristic of these conditions.

Iron is a vital nutrient that performs multiple roles in cellular processes, ranging from DNA replication and cell growth to protection against oxidative stress. In the healthy host, the majority of iron is bound with intracellular proteins and the remaining free iron is extracellular and insoluble, and hence difficult to access [88]. For invading pathogens, siderophore systems are critical for iron competition and uptake to accomplish colonization and cause infections, and this is particularly true in the preterm GI tract. Preterm infants in NICU are heavily supplemented with iron as they receive many red blood cell transfusions (increasing hepatic iron stores), iron-supplemented parenteral nutrition and supplementary oral iron within a few weeks of birth. During infection, *Klebsiella* secretes siderophores to sequester iron and establish colonization in the host. Enterobactin is the most well-known siderophore produced by *K. pneumoniae* and related species, and was found to be encoded by all our strains (Figs 2b and 3b). The host innate immune protein lipocalin 2 binds to enterobactin and disrupts bacterial iron uptake [89]. *Klebsiella* species have evolved to hoodwink this host response by producing several evasive siderophores [90, 91]. Siderophore production of *Klebsiella* isolates was monitored using the CAS liquid assay. All isolates tested grown in M9 minimal medium were CAS-positive, with the estimated siderophore concentration ranging between of 3.5 and 6 nM (Fig. 4b). There was no significant difference in siderophore production between healthy and NEC- and sepsis-associated isolates.

*Klebsiella* is of concern within an AMR context, particularly in at-risk neonates, due to the increasing emergence of multidrug-resistant isolates that cause severe infection [92]. A UK study in which 24 % of all LOS cases were caused by *Enterobacteriaceae* (8.9 % of all caused by *Klebsiella* spp.) showed that a high proportion (14 and 34 %, respectively) of *Enterobacteriaceae* isolates recovered from sick infants were resistant to flucloxacillin/gentamicin and amoxicillin/cefotaxime, the two most commonly used empirical antibiotic combinations [51]. Thus, to demonstrate antibiotic resistance phenotypes in *Klebsiella* spp. correlating to presence of AMR genotypes, we tested the susceptibility of the isolates with three antibiotics commonly prescribed in NICUs; gentamicin, meropenem and benzylpenicillin (Table 1). One strain of *K. grimontii* (#80) was potentially sensitive to benzylpenicillin, an aminopenicillin currently not recognized as being clinically useful against *Enterobacteriaceae *.

**Table 1.** Determination of MICs for *Klebsiella* spp. isolates

Isolate ID	Species	Gentamicin (mg l^−1^)*	Meropenem (mg l^−1^)†	Benzylpenicillin (mg l^−1^)‡
#64	* K. pneumoniae *	**6.25§**	3.13	1560
#74	* K. pneumoniae *	1.5625	6.25	3130
#85	* K. pneumoniae *	1.5625	3.13	3130
#91	* K. quasipneumoniae *	1.5625	6.25	3130
#92	* K. pneumoniae *	1.5625	3.13	3130
#95	* K. pneumoniae *	3.125	3.13	3130
#102	* K. pneumoniae *	1.5625	6.25	3130
#115	* K. pneumoniae *	1.5625	3.13	3130
#118	* K. pneumoniae *	1.5625	6.25	3130
#80	* K. grimontii *	3.125	1.56	6.25
#83	* K. grimontii *	**12.5**	1.56	780
#99	* K. grimontii *	3.125	0.78	780
#88	* K. michiganensis *	3.125	3.13	3130
#108	* K. michiganensis *	**6.25**	1.56	3130

* *Enterobacteriaceae* EUCAST breakpoint for gentamicin resistance is >4 mg l^−1^, and for sensitivity it is ≤2 mg l^−1^.

†*Enterobacteriaceae* EUCAST breakpoint for meropenem resistance is >8 mg l^−1^, and for sensitivity it is ≤2 mg l^−1^.

‡No *Enterobacteriaceae* EUCAST data are available for benzylpenicillin.

§Bold type, resistant; underlined, intermediate.

Isolates #64, #83 and #108 (all encoding KpnG and KpnH; Figs 2a and 3b) were resistant to gentamicin, while #80, #88, #95 and #99 (all encoding KpnG and KpnH; Figs 2a and 3b) showed intermediate susceptibility to this aminoglycoside.

The presence of a gene in a bacterium’s genome does not mean it is functionally active, and nor does it give any indication as to how active the gene is if it is indeed functional; e.g. all nine *K. pneumoniae* isolates encoded KpnG and KpnH (strict CARD matches; Fig. 2a), but only two showed any resistance to gentamicin upon susceptibility testing.

Isolates #64 (SHV-1), #74 (SHV-36), #85, #88, #91 (OKP-A-2), #92 (SHV-36), #95 (SHV-28), #102 (SHV-164), #115 (SHV-36) and #118 (SHV-36) – which all encoded extended-spectrum β-lactamases (SHV) or *Escherichia ampH* β-lactamase (OKP-A-2) but lacked OmpK35 and OmpK36 – showed intermediate susceptibility to the carbapenem meropenem. Loss of the two porins OmpK35 and OmpK36 is known to confer resistance to carbapenems in strains producing extended-spectrum β-lactamases or plasmid-mediated AmpC-type β-lactamases [93]. *K. pneumoniae* #64 was isolated from an infant with clinically diagnosed NEC with confirmed *Klebsiella* colonization. Importantly, this preterm infant had previously been treated with benzylpenicillin, gentamicin and meropenem, which may link to the observed phenotypic resistance and corresponding AMR genes *Escherichia ampH* β-lactamase, *bla*_SHV-1_ and KpnG/KpnH and SHV-1, and lack of OmpK35 and OmpK36, and suggests further treatment with gentamicin and meropenem would have been ineffective in this infant. Indeed, the infant was treated with cefotaxime, metronidazole and vancomycin in a subsequent round of medication (Table S2). *K. pneumoniae* #115 was isolated from a baby that had confirmed NEC: the strain was resistant to benzylpenicillin (encoded *Escherichia ampH* β-lactamase and *bla*_SHV-36_) and showed intermediate resistance to meropenem (lacked OmpK35 and OmpK36). *K. michiganensis* #88, also isolated from a baby that had NEC, showed intermediate resistance to both benzylpenicillin (*bla*_OXY-1-2_, perfect CARD match; Fig. 3b) and meropenem (lacked OmpK35 and OmpK36): both antibiotics had been administered to the baby at birth. *K. grimontii* #99, isolated from a baby with suspected sepsis, showed intermediate resistance to benzylpenicillin (encoded *Escherichia ampH* β-lactamase and *bla*_OXY-6-2_). These data indicate that preterm-associated *Klebsiella* have a multidrug-resistant phenotype that may prove problematic when treatment options are required for sepsis or NEC. Interestingly, other isolates (e.g. #95, recovered from an infant who had received benzylpenicillin and gentamicin; Table S2) associated with healthy preterm infants also harboured AMR genes (#95: Fig. 2a) and phenotypic resistance profiles suggesting that administration of antibiotics to preterm infants with no signs of clinical infection contributes to the reservoir of AMR genes – the ‘resistome’ [94] – which may increase horizontal gene transfer of AMR determinants to other opportunistic pathogens residing within the GI tract.

### Abundance of *K. oxytoca* and related species in metagenomic datasets

We used a published metagenomics dataset [1] to determine the prevalence of *K. oxytoca *, *K. michiganensis* and *K. grimontii* in the preterm infant gut microbiome. These data had previously been used to look at the relationship between NEC and uropathogenic *E. coli *, and metadata were available for the samples. Ward *et al.* [1] collected a total of 327 samples at 3 stages of infant life: stage 1, days 3–9; stage 2, days 10–16; stage 3, days 17–22. Within each life stage, samples were collected on more than 1 day for some infants. In the current study, only samples processed under protocol A of Ward *et al.* [1] and from the earliest collection day within each life stage were analysed. For those samples for which multiple sets of paired-end data were available, read data were concatenated and used in analyses (Table S3).

Stage 1 comprised samples from 127 infants (105 preterm, 22 term), 16 of whom had been diagnosed with NEC with 10 infants having subsequently died. Stage 2 comprised samples from 146 infants (128 preterm and 18 term), 24 of whom later developed NEC with 18 deaths. Stage 3 comprised samples from 54 infants (48 preterm, 6 term), including 8 NEC patients, 6 of whom died. Samples were collected from 165 distinct infants (143 preterm, 22 term), but only 41 of them were sequenced at all 3 life stages. Infants were born either vaginally (*n*=70) or by caesarean section (*n*=95). The gestational ages of preterm infants ranged from 23 to 29 weeks (mean 26.1 weeks), while the term babies ranged from 38 to 41 weeks (mean 39.2 weeks).

**Fig. 5.**
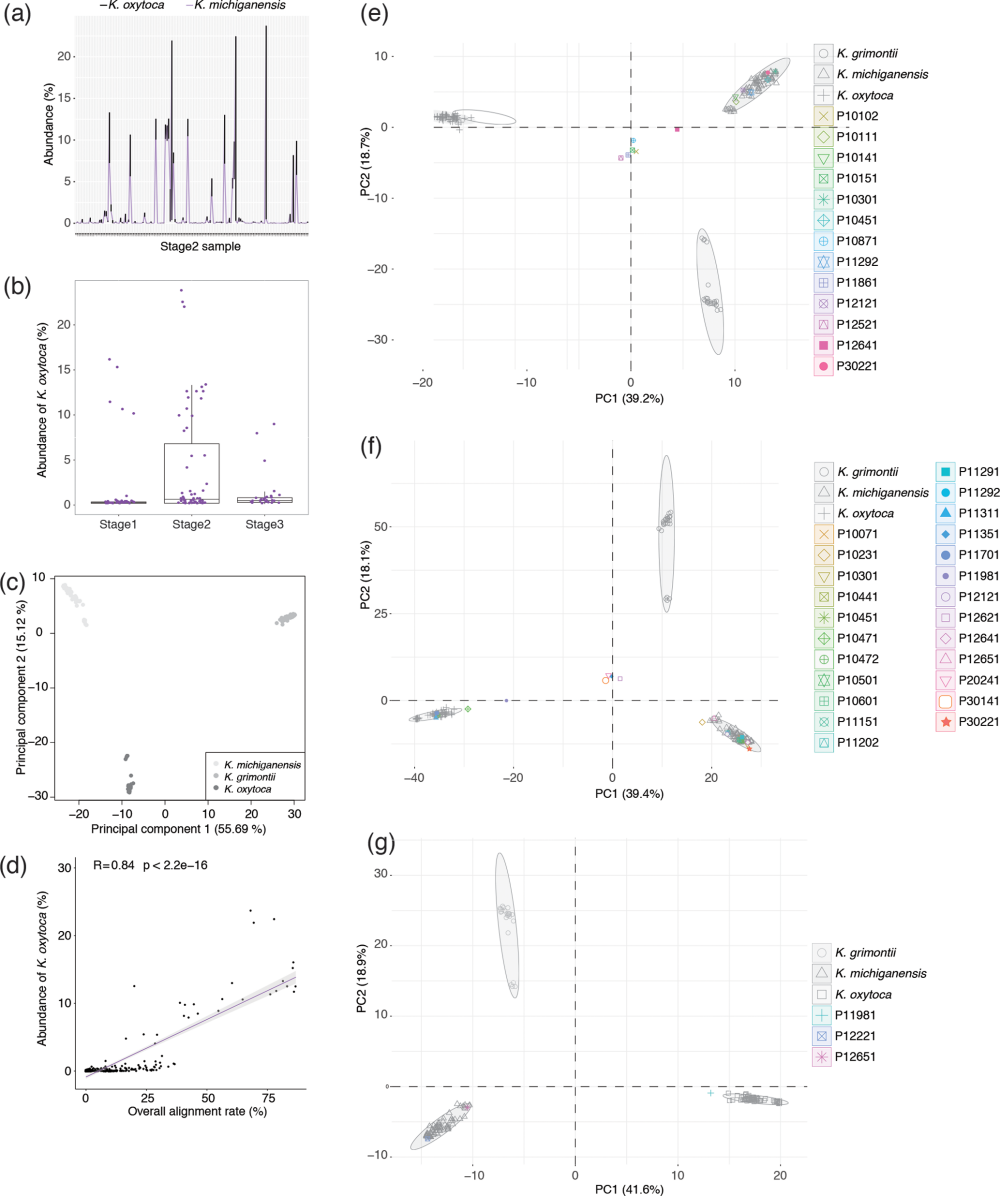
Identification of *K. oxytoca *-related species in infant faecal metagenomes. (a) Comparison of *K. oxytoca* and *K. michiganensis* abundance (as determined using Centrifuge) in the stage 2 samples of Ward *et al.* [1]. (b) Abundance of *K. oxytoca* (determined using Centrifuge) across stage 1, stage 2 and stage 3 samples of Ward *et al.* [1]. (c) Separation of the strains of *K. grimontii* (*n*=27), *K. michiganensis* (*n*=76) and *K. oxytoca* (*n*=64) based on accessory genes (*n*=5,108) detected in the Roary-generated open pangenome. (d) Relationship between PanPhlAn (overall alignment rate) and Centrifuge [abundance *K. oxytoca* (%)] data. (e, f, g) PCA plots show separation of strains in the pangenome plus PanPhlAn-detected strains based on the presence of 500 randomly sampled accessory genes at (e) stage 1, (f) stage 2 and (g) stage 3 of Ward *et al.* [1].

As we had found that the MetaPhlAn2.6 database contained non-* K. oxytoca* genomes within its *K. oxytoca* dataset [detecting *K. oxytoca *, *K. michiganensis* and *R. ornithinolytica* (refer to the Methods section)], we used Centrifuge to determine the abundance of this species in metagenomes (Fig. 5a, b). Due to their genomic similarity, *K. oxytoca* and *K. michiganensis* could not be readily distinguished using Centrifuge (Fig. 5a); no genomes assigned to *K. grimontii* were included in the Centrifuge database at the time this study was undertaken. Although it should be noted that, while Centrifuge (and Kraken2) relies on NCBI taxonomy for species identification, there are still many genomes within GenBank/RefSeq that are assigned to the wrong species [e.g. assemblies GCA_001052235.1 and GCA_000427015.1 within our curated pangenome dataset have been confirmed by detailed analyses to be *K. grimontii* (Fig. S2 and Chen [36]), but were still assigned as *K. oxytoca* and *K. michiganensis *, respectively, within GenBank as of 28 July 2019; these are by no means the only examples from our current study].

For those samples harbouring *K. oxytoca *, the relative abundance of the bacterium increased from stage 1 to stage 2 and decreased at stage 3 (Fig. 5b).

### Metapangenome analysis of preterm infant metagenomic data to detect *K. oxytoca *, *K. michiganensis* and *K. grimontii*

Using a set of 162 *K. oxytoca *-related genomes (Table S4) and those of the 5 infant isolates, a pangenome was generated using Roary. The pangenome dataset consisted of 76 *K. michiganensis* [mean ANI among strains 98.55 (0.60) %, range 97.13–100 %], 64 *K. oxytoca* [mean ANI among strains 99.20 (0.30) %, range 98.53–100 %] and 27 *K. grimontii* [mean ANI among strains 98.45 (1.47) %, range 95.70–100 %] strains. A total of 40 605 genes were detected in the open pangenome: 2769 of them constituted the core gene cluster, while the accessory cluster included 5108 genes and the remaining 32 728 genes formed the strain-specific cluster. A PCA plot based on the accessory genes clustered strains into the three different species (Fig. 5c), in agreement with our phylogenetic analysis of the core genes (Fig. S5b) and consistent with the findings of Moradigaravand *et al*. [76], who were able to split the three species (phylogroups) based on a pangenome analysis of fewer genomes.

The Roary-generated pangenome was used as a custom database for PanPhlAn, to detect the presence and absence of core genes and accessory genes in each infant sample. As expected, the proportion of reads that PanPhlAn mapped to the custom database correlated with the Centrifuge-generated abundance data (Fig. 5d). In stage 1, 13 infants (12 preterm, 1 term; 10.2 % of all stage 1 samples) were predicted to carry *K. oxytoca *-related species (Fig. 5e); in stage 2, the number was 24 (22 preterm, 2 term; 16.4 % of all stage 2 samples) (Fig. 5f); and in stage 3 infants, the rate of carriage was much lower, with only 3 infants (all preterm; 5.6 % of all stage 3 samples) potentially harbouring target species (Fig. 5g). The change in prevalence of *K. oxytoca *-related species across the three stages based on the PanPhlAn analysis was consistent with the *K. oxytoca* abundance data generated with Centrifuge (Fig. 5b).

The pangenome accessory genes were used in PCA to define which species strains detected by PanPhlAn belonged to. In stage 1, samples from 6 preterm infants (P10111, P10141, P10301, P10451, P11292, P12121) and 1 term infant (P30221) harboured *K. michiganensis* (Fig. 5e). In stage 2, samples from 4 preterm infants (P10471, P10472, P11311, P11701) harboured *K. oxytoca *, while those from 14 preterm infants (P10071, P10231, P10301, P10441, P10451, P10501, P10601, P11151, P11202, P11291, P11292, P12121, P12641, P12651) and 1 term infant (P30221) harboured *K. michiganensis* (Fig. 5f). Four samples (P11351, P12621, P20241, P30141) could not be assigned a species, while the sample from P11981 located close to *K. oxytoca* (Fig. 5f). Similarly, 2 of the stage 3 samples from P12221 and P12651 carried *K. michiganensis *, while P11981 located near *K. oxytoca* (Fig. 5g).

### Recovery of *K. oxytoca* and *K. michiganensis* MAGs from metagenomes

Since the abundance of *K. oxytoca *-related species was considerable in some infant samples, we attempted to obtain high-quality MAGs directly from these metagenomes and to assign them to the species. The metagenomic samples were checked and their reads aligned against those of the 167-genome database; reads that mapped were extracted and assembled as ‘original MAGs’. The genome sizes of the 167 genomes ranged from 5.72 to 7.23 Mb (mean 6.35 Mb), and thus a genome size of at least 5.5 Mb was used to define a likely complete MAG. After assembly of the reads that mapped to our database, MAGs were generated from the stage 1 (_s1), stage 2 (_s2) and stage 3 (_s3) samples. ANI and phylogenetic analyses showed these MAGs to be *K. michiganensis* (P10301_s1, P10451_s1, P11292_s1, P12121_s1, P30221_s1, P10071_s2, P10301_s2, P10441_s2, P10451_s2, P10501_s2, P10601_s2, P11151_s2, P11202_s2, P11291_s2, P11292_s2, P12121_s2, P12641_s2, P12651_s2, P30221_s2, P12221_s3 and P12651_s3; mean ANI with GCA_002925905 of 98.61±0.66 %) or *K. oxytoca* (P10472_s2, P11311_s2, P11701_s2, P11981_s2; mean ANI with GCA_900083895 of 99.30±0.26 %) [36]. No *K. grimontii* MAGs were recovered from any samples.

Prior to checking the completeness and contamination of the *K. michiganensis* and *K. oxytoca* MAGs, contigs <500 nt in length were removed from the assemblies. A high-quality MAG requires a >90 % genome completeness with contamination <5 % [44]. According to CheckM results, 3 stage 1 MAGs (P10301_s1, P10451_s1, P30221_s1), 7 stage 2 MAGs (P10441_s2, P10451_s2, P10501_s2, P10601_s2, P11291_s2, P11292_s2, P30221_s2) and 1 stage 3 MAG (P12221_s3) were of high quality. The rest of the MAGs were ≥90 % complete, but were contaminated (e.g. P12621_2 contained 274.59 % contamination). Thus, we attempted to decontaminate the MAGs using a Diamond blast-based approach.

Since we already knew the species each MAG belonged to from PCA and PanPhlAn analyses, scaffolds were mapped against the relevant species-specific genome database under different minimum blast identities to report alignments, which were used to generate ‘cleaner’ MAGs. Fig. S6 shows the change in genome completeness and the percentage contamination of stage 2 MAGs when different blast identities were applied. The changes were negligible for those MAGs with high-quality-level completeness and lacking contaminants even when the cut-off was set at 99 %. For contaminated MAGs, the percentage contamination decreased markedly as the blast identity became stricter and reduced to the bottom when all scaffolds in that MAG could be aligned with 100 % identity. However, 100 % was not an appropriate threshold as the genome completeness was affected greatly at this point (Fig. S6). Instead, a cut-off of 99 % was used to decontaminate MAGs because 13 high-quality level MAGs and 5 medium-quality level MAGs could be obtained when using this identity threshold. Stage 2 MAGs that passed PanPhlAn, PCA and ANI analysis reached at least reach medium-quality level using a 99 % identity threshold. This cut-off was also suitable for stage 1 MAGs, the quality of which was high. However, for stage 3 MAGs, the percentage contamination from P11981_s3 only decreased to medium-quality level at 100 % identity, at which time the genome completeness fell down to 42.40 %. After evaluating their genome completeness and contamination levels, a total of 25 MAGs (Table 2) were assessed further.

**Table 2.** Summary statistics for MAGs recovered from preterm infant metagenomes

MAG*	Genome length (bp)	Max. contig length	Coverage†	N50	No. of scaffolds	GC content (%)	Completeness (%)‡	Contamination (%)‡	CDS	No. of tRNAs	No. of rRNAs	Species	Quality	*gapA*	*infB*	*mdh*	*pgi*	*phoE*	*rpoB*	*tonB*	ST
10071_s2	6 577 866	334 901	~131×	70 229	349	52.95	97.59	3.64	6204	54	3	* K. michiganensis *	High	3	9	8	9	20	*	8	*
10301_s1	6 304 211	308 355	~158×	143 120	176	53.15	99.70	0.48	5920	54	4	* K. michiganensis *	High	3	5	21	13	74	6	12	202
10301_s2	6 504 340	160 223	~21×	30 917	609	54.93	98.69	4.21	6191	50	2	* K. michiganensis *	Medium	3	5	21	13	74	6	12	202
10441_s2	6 128 395	396 555	~85×	122 210	176	53.35	100.00	0.71	5691	58	4	* K. michiganensis *	High	3	5	21	13	24	6	*	*
10451_s1	6 135 593	499 885	~79×	130 343	154	53.44	100.00	0.71	5708	53	7	* K. michiganensis *	High	3	5	21	13	24	6	*	*
10451_s2	6 140 759	377 549	~65×	130 433	166	53.53	100.00	0.71	5707	54	4	* K. michiganensis *	High	3	5	21	13	24	6	*	*
10472_s2	6 352 656	271 779	~46×	80 647	252	53.26	99.90	2.59	5889	50	3	* K. oxytoca *	High	1	7	2	1	65	1	2	176
10501_s2	6 179 579	451 918	~39×	133 209	217	54.01	100.00	1.34	5730	55	9	* K. michiganensis *	High	3	5	21	3	24	6	*	*
10601_s2	6 014 627	328 826	~59×	181 417	121	52.63	99.96	0.34	5540	56	4	* K. michiganensis *	High	3	5	21	3	24	6	*	*
11151_s2	6 595 368	114 676	~40×	17 470	949	54.71	97.17	5.81	6252	59	0	* K. michiganensis *	Medium	3	5	21	13	20	*	12	*
11202_s2	6 328 179	267 673	~144×	111 043	224	53.38	98.81	3.22	5864	54	3	* K. michiganensis *	High	*	8	24	33	20	6	23	*
11291_s2	6 301 261	429 108	~45×	146 444	193	53.10	99.70	0.79	5911	68	8	* K. michiganensis *	High	3	8	17	21	40	17	29	84
11292_s1	6 533 348	267 672	~43×	118 981	400	53.17	98.81	2.77	6064	48	3	* K. michiganensis *	High	*	8	24	33	20	6	23	*
11292_s2	6 383 200	267 500	~70×	119 207	202	50.77	99.70	2.49	5891	63	8	* K. michiganensis *	High	*	8	24	33	20	6	23	*
11311_s2	6 427 314	266 393	~137×	66 881	516	52.89	99.97	2.96	5986	46	1	* K. oxytoca *	Medium	2	2	2	3	19	2	2	199
11701_s2	6 230 855	162 114	~60×	50 736	555	54.40	99.85	3.20	5807	50	2	* K. oxytoca *	Medium	1	7	2	1	65	1	2	176
11981_s2	5 658 923	237 765	~10×	60 298	326	53.62	89.43	2.20	5280	30	2	* K. oxytoca *	Medium	*	2	2	3	19	*	2	*
12121_s1	5 843 313	426 086	~121×	101 108	251	54.02	96.40	2.49	5358	53	4	* K. michiganensis *	High	3	33	17	45	20	6	48	149
12121_s2	5 690 611	180 068	~82×	56 100	371	54.36	94.21	2.85	5226	35	3	* K. michiganensis *	High	3	33	17	45	20	*	48	*
12221_s3	6 115 156	469 104	~17×	174 402	147	54.67	99.70	1.60	5667	63	8	* K. michiganensis *	High	3	5	21	20	24	6	30	108
12641_s2	6 383 005	237 677	~55×	80 508	279	53.91	99.44	3.15	6027	56	2	* K. michiganensis *	High	3	5	21	13	74	*	12	*
12651_s2	5 124 388	199 319	~28×	81 423	207	53.59	94.05	3.21	4801	42	3	* K. michiganensis *	High	14	24	15	8	18	*	4	*
12651_s3	5 422 817	363 696	~10×	73 253	207	53.78	95.49	2.40	5047	41	2	* K. michiganensis *	Medium	14	2	15	8	18	*	4	*
30221_s1	6 220 970	480 926	~51×	173 840	132	52.47	99.70	1.42	5770	61	9	* K. michiganensis *	High	3	5	21	20	11	6	20	43
30221_s2	6 224 584	349 605	~55×	170 314	142	52.27	99.70	1.39	5765	58	7	* K. michiganensis *	High	3	5	21	20	11	6	20	43

*E.g. 10301_s1 represents a MAG recovered from infant 10301 at stage 1.

†Coverage, based on coverage of longest contig (determined from SPAdes data).

‡Completeness and contamination determined using CheckM (v1.0.18).

The presence of tRNAs for the standard 20 amino acids and rRNA was examined as a secondary measure of genome quality. A high-quality MAG requires at least 18 of the 20 possible amino acids [44]. P11981_s2 (16 aa) and P12651_s3 (17 aa) had to be classified as medium-quality MAGs, even though their genome completeness and contamination reached high-quality levels. 16S rRNA genes were detected in all MAGs except P11151_s2, which was subsequently classified as medium quality. Taking mandatory genome information into consideration [44], a total of 19 high-quality and 6 medium-quality MAGs were recovered (Table 2); the sequences of these MAGs are available from figshare (https://figshare.com/articles/MAGs_zip/11923005). All of the MAGs had ≥15 standard tRNAs. High-quality MAGs had tRNAs that encoded an average of 19.6 (0.7) of the 20 amino acids, and some of them even had a tRNA that encodes an additional amino acid SeC, while medium-quality MAGs had 18 (1.4) basic amino acids encoded by tRNAs. High-quality MAGs consisted of ≤500 scaffolds in 52.6 % of cases (mean 600) and had an average N50 of 121 kb, while only one medium-quality MAG comprised ≤500 scaffolds (mean 1174) and the average N50 was less than half of that of high-quality MAGs (48.3 kb).

### Genotyping of MAGs

**Fig. 6.**
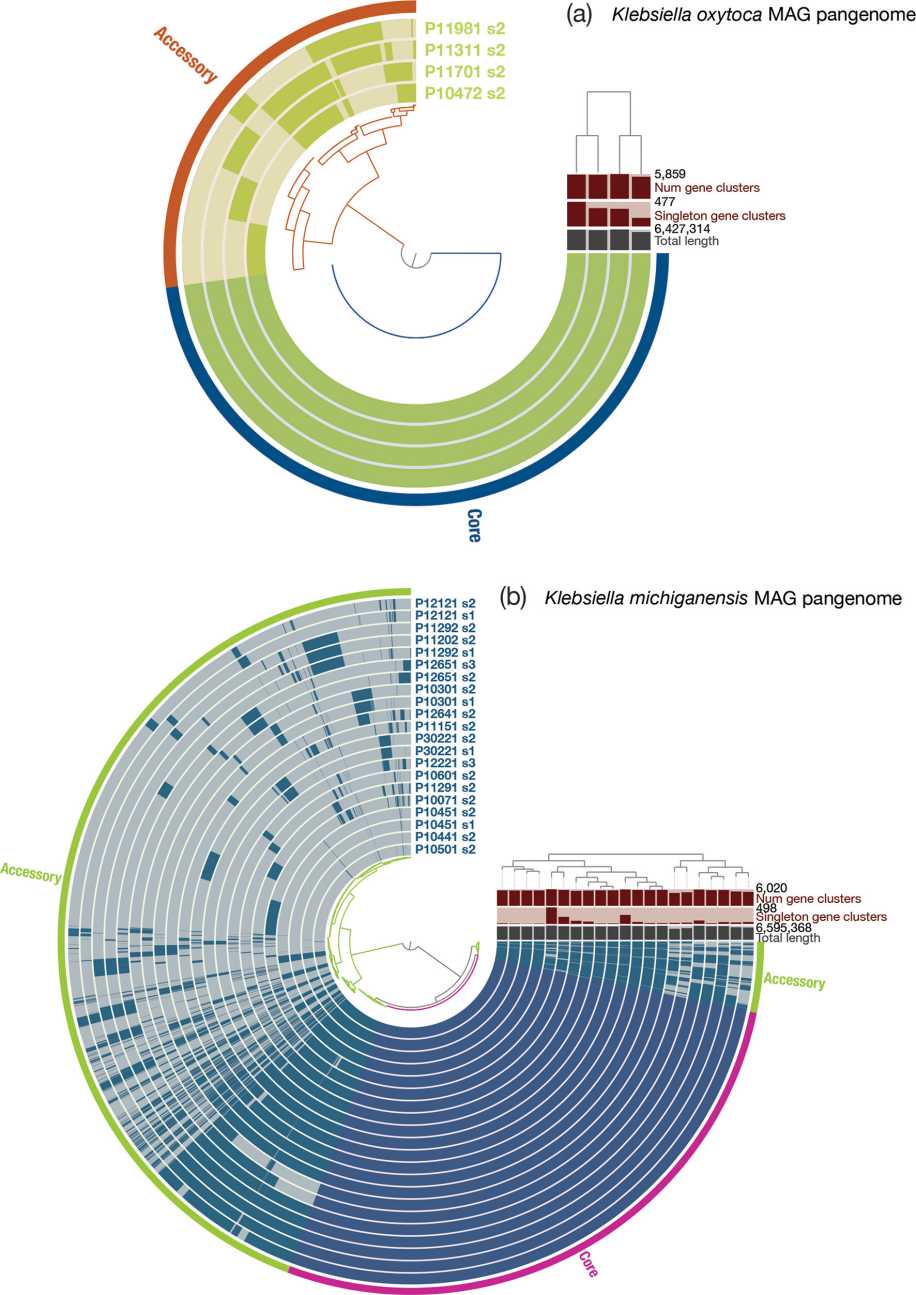
Anvi’o representation of the MAGs recovered from the metagenomes of infants included in the study of Ward *et al.* [1]. (a) *K. oxytoca *. (b) *K. michiganensis *. It is notable that MAGs recovered from different life stages from the same infant (e.g. 10301_s1, 10301_s2) are highly similar to one another.

Comparison of the sequences of the MAGs showed that each infant harboured a different strain of *K. oxytoca* (Fig. 6a) or *K. michiganensis* (Fig. 6b). In infants where MAGs were recovered across different life stages, the MAGs were highly similar to one another (Fig. 6b). Similar to what we had seen with our isolates, the MAGs encoded a range of β-lactamase and virulence genes (Fig. S7). It was also notable that two of the MAGs (* K. michiganensis* 10071_s2, *K. oxytoca* 10472_s2) encoded *mcr-9* (perfect match), a plasmid-mediated colistin resistance gene and phosphoethanolamine transferase. However, as noted above for our isolate work, the presence of the aforementioned genes in MAGs does not mean they were functionally active in the infants’ GI tracts. All the *K. michiganensis* MAGs encoded the siderophore enterobacterin, along with all but one (11981_s2) of the *K. oxytoca* MAGs. The allantoinase gene cluster associated with liver infection was detected in the four *K. oxytoca* MAGs, but only a third of the *K. michiganensis* MAGs. We only detected this cluster in the *K. grimontii* isolates we recovered (Fig. 3b).

MLST analysis assigned 10 MAGs to 8 known STs (Table 2). We believe insufficient sequence coverage meant we were unable to ST more MAGs: e.g. *gapA* of MAG P11981_s2 aligned exactly with the *gapA* allele 2 sequence, but it was only partial, leaving an incomplete match.

The STs of all genomes in the curated genome dataset were also identified (Table S4). Due to our limited understanding of *K. oxytoca *-related species, many combinations of alleles have not been assigned corresponding STs yet, especially for the newly described species *K. grimontii* [73]. MLST identification showed that 54/64 *K. oxytoca sensu stricto* strains had known STs, with some STs being more dominant than others. ST2, the most prevalent ST and represented by 15 strains, belonged to CC2. ST18 and ST19 were also in CC2, with both represented by two strains. ST199 and ST176 were the second and third most common STs, respectively. Among *K. michiganensis* strains, 48/76 could be assigned a known ST and comprised 21 distinct STs, with 9 represented by more than 1 isolate. ST11, ST27, ST50, ST85, ST143 and ST202 were the most frequent STs, all of which were represented by at least four strains. *K. michiganensis* #108 was ST157. Only 5/27 *K. grimontii* strains could be assigned an ST (#83 – ST72; #99 – ST76; 10–5250 – ST47, GCA_000247915.1; 1148_KOXY – ST186, GCA_001052235.1; M5al – ST104, GCA_001633115.1).

## Summary

*Klebsiella* spp. encode numerous virulence and antibiotic resistance genes that may contribute to the pathogenesis of NEC and LOS. In this study, we characterized nine *K. pneumoniae *, three *K. grimontii* and two *K. michiganensis* strains isolated prospectively from the faeces of a UK cohort of preterm infants, and have shown these gut isolates are able to reside and persist in macrophages, suggesting they can evade the immune system. These isolates will be used in future studies aiming to replicate aspects of NEC and sepsis in model systems to confirm the role of *Klebsiella* spp. in these diseases.

We have shown that misannotated genomes are being used in bioinformatics tools routinely used to characterize the human gut microbiome. By using a carefully curated dataset to undertake metapangenome analyses of the closely related species *K. oxytoca *, *K. michiganensis* and *K. grimontii *, we have demonstrated that *K. michiganensis* is likely to be more clinically relevant to a subset of preterm infants than *K. oxytoca *. The identity of publicly available genomes should be confirmed upon download and linked to accurate taxonomic frameworks prior to analyses of data, especially when attempting to identify and type closely related species in metagenomic data.

## Data Bibliography

1. 16S rRNA gene sequence data have been deposited at DDBJ/ENA/GenBank under BioProject accession PRJEB34372.

2. The Whole Genome Shotgun project has been deposited at DDBJ/ENA/GenBank under BioProject accession PRJNA471164.

3. The metagenome-assembled genomes are available from figshare (https://figshare.com/articles/MAGs_zip/11923005).

## Supplementary Data

Supplementary material 1

Supplementary material 2
