# Supplementary material for: Preterm infants harbour diverse Klebsiella populations, including atypical species that encode and produce an array of antimicrobial resistance- and virulence-associated factors
Source: Microb Genom. 2020 May 21;6(6):e000377. doi: 10.1099/mgen.0.000377 (PMC7371107; doi:10.1099/mgen.0.000377)
Supplement: Supplementary material 1 [file mgen-6-377-s001.pdf]

**Supplementary Figures associated with Chen *et al.* – Preterm infants harbour diverse *Klebsiella* populations, including atypical species that encode and produce an array of antimicrobial resistance- and virulence-associated factors**

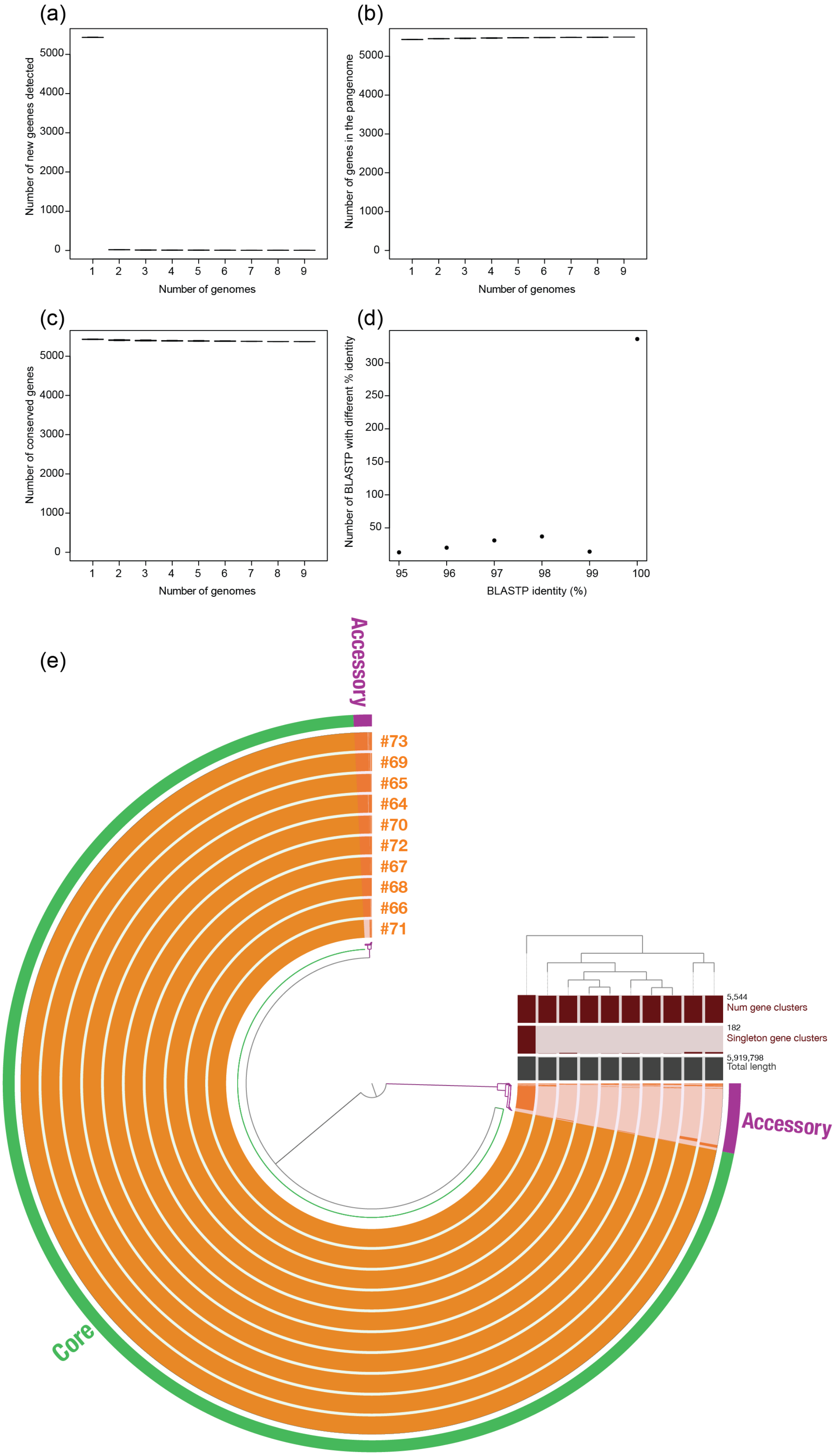

(a)
Number of new geenes detected
5000
4000
3000
2000
1000
0
1 2 3 4 5 6 7 8 9
Number of genomes
(b)
Number of genes in the pangenome
5000
4000
3000
2000
1000
0
1 2 3 4 5 6 7 8 9
Number of genomes
(c)
Number of conserved genes
5000
4000
3000
2000
1000
0
1 2 3 4 5 6 7 8 9
Number of genomes
(d)
Number of BLASTP with different % identity
300
250
200
150
100
50
95 96 97 98 99 100
BLASTP identity (%)
(e)
Accessory
#73
#69
#65
#64
#70
#72
#67
#68
#66
#71
5,544
Num gene clusters
182
Singleton gene clusters
5,919,798
Total length
Accessory
Core

**Supplementary Figure 1.** Genomic characterization of nine *K. pneumoniae* isolates recovered from neonate P008. (a) Change in number of new genes detected as the number of genomes increased. The number of new genes detected fell to almost 0 when a second genomes joined the first, and stayed at around 0 as further genomes were added to the analyses. (b, c) Change in total number of genes and conserved genes in the pangenome. As the number of genomes increased, the number of total genes (b) and conserved genes (c) is almost stable, with only negligible changes observed. (d) Number of BLASTP hits at different percentage identity. Identities of all hits are over 95 %, with the majority of them 100 % identical, providing more evidence to show the isolates from neonate P008 share the same genetic content. (e) Anvi'o representation of genomes of the isolates recovered from neonate P008. Differences between the isolates are due to the presence of single gene clusters in genomes, considered to be due to differences in genome coverage across the isolates.

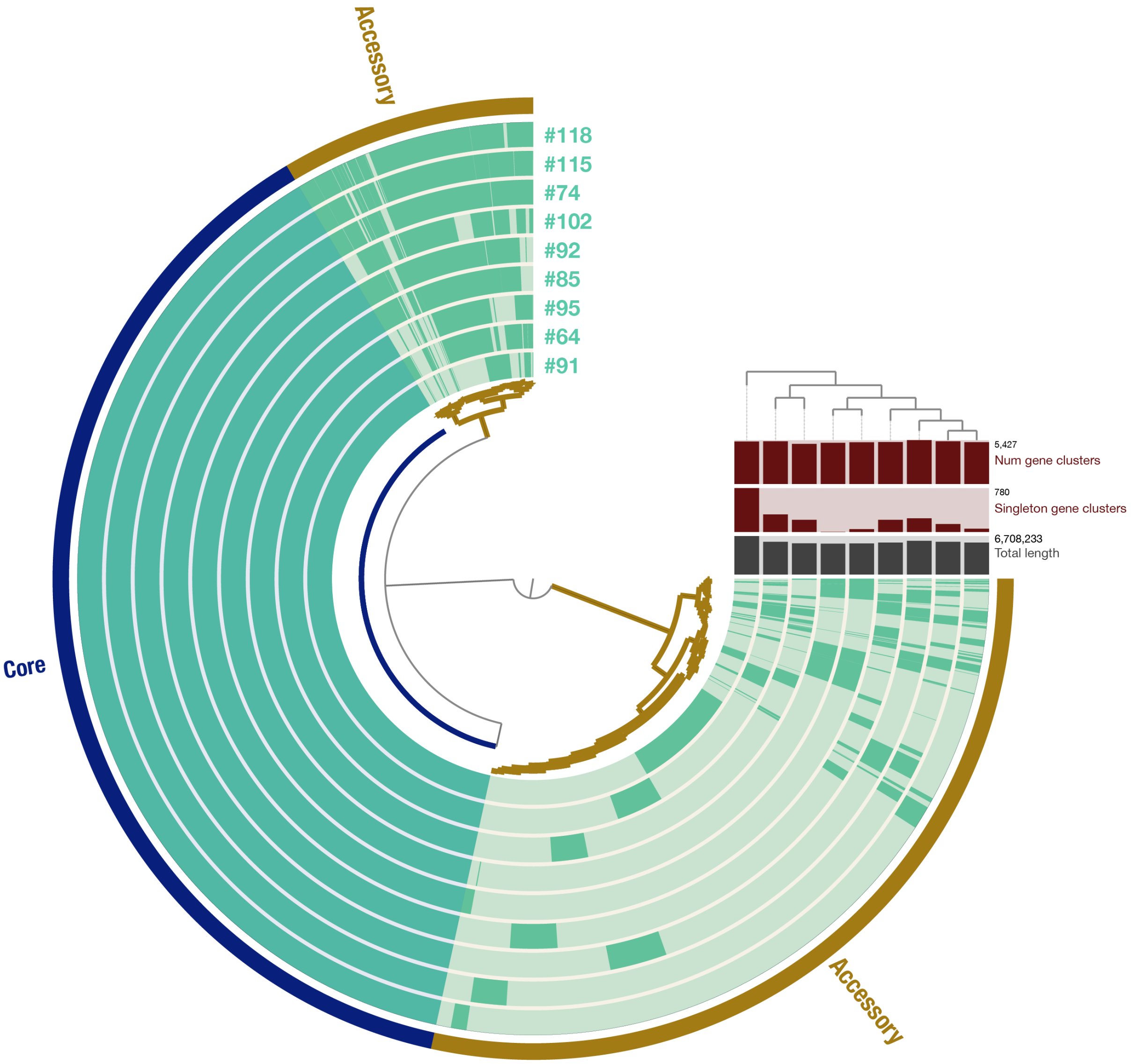


**Supplementary Figure 2.** Anvi'o representation of genomes of the *K. pneumoniae* isolates recovered from different infants. The genomes are clearly different from one another, indicating each isolate represents a different strain of *K. pneumoniae*.

(a)

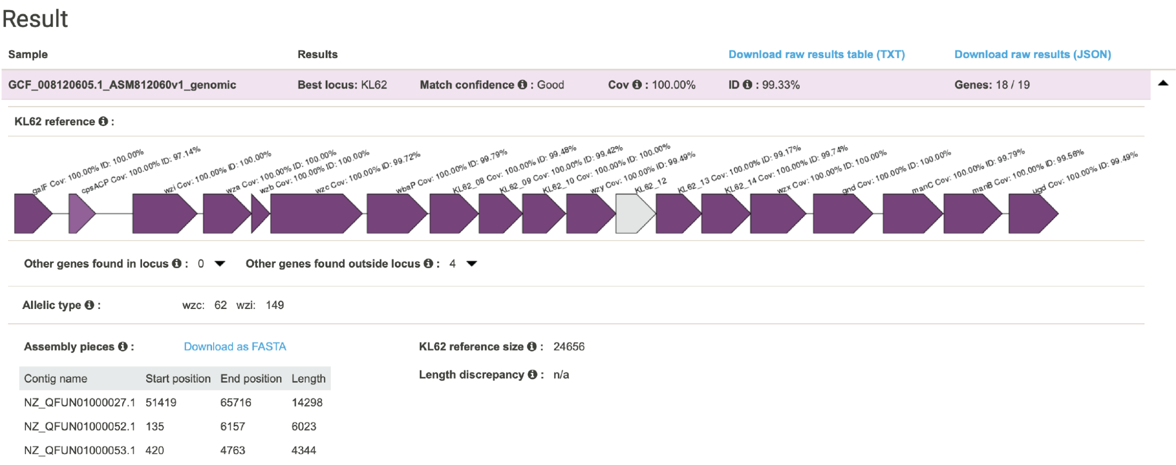


(b)

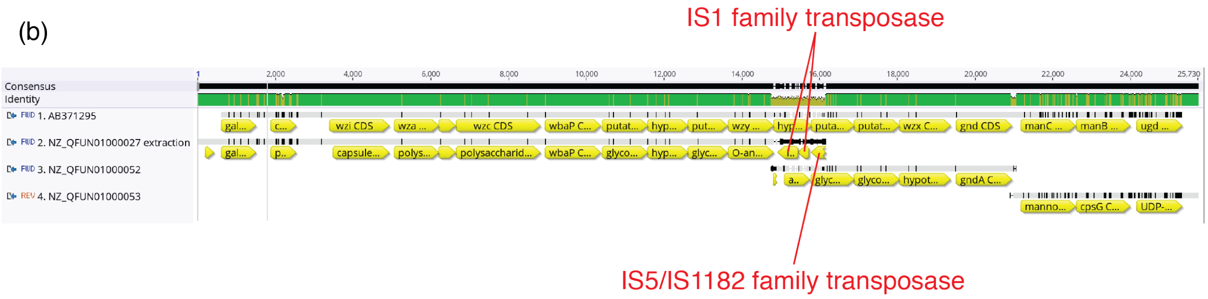


**Supplementary Figure 3.** Isolate #74 does not have an intact set of K62 capsular genes. (a) Analysis of the isolate's genome using Kaptive showed hits across three contigs and suggested the isolate encoded a variant of the K62 capsule type. (b) Comparison of the Kaptive K62 reference sequence (GenBank accession number AB371295) with the three contigs of #74 showed the K62 genes are non-contiguous. The ClustalW 2.1 alignment was created using Geneious Prime v2019.2.1.

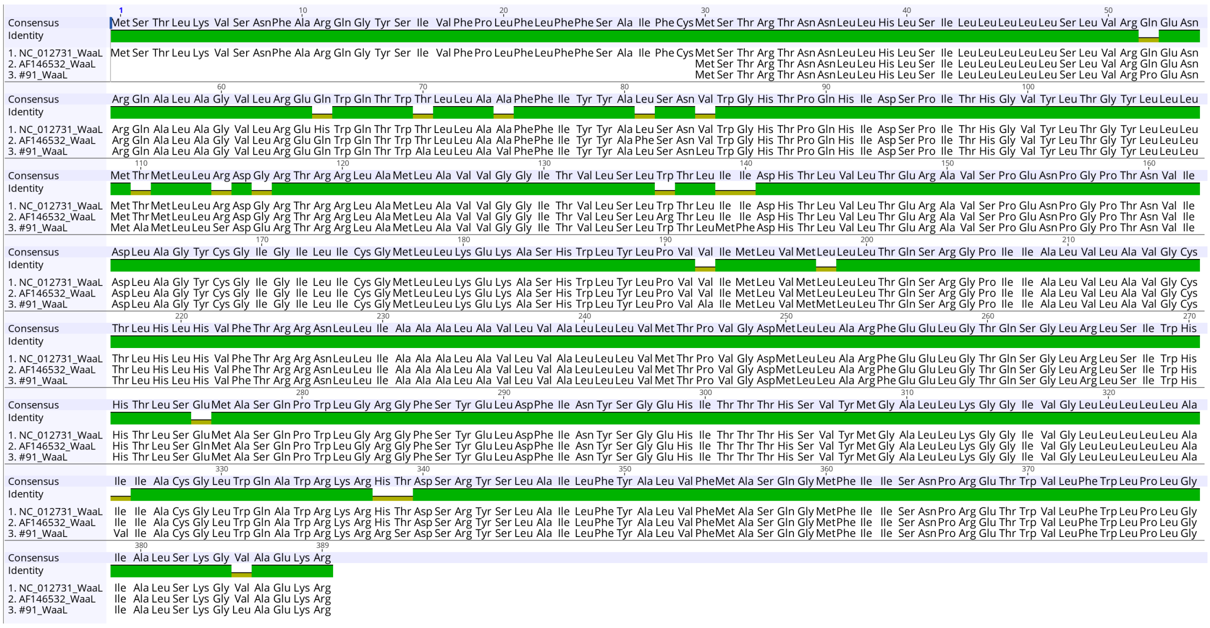

**Supplementary Figure 4.** Demonstration that *K. quasipneumoniae* #91 K11:O3 encodes a type 1 LPS core. The WaaL-encoding sequence of #91 was identified by comparison of the genome of #91 with the *waa* gene cluster described by Regué *et al.* (66) (GenBank accession number AF146532). WaaL of *K. quasipneumoniae* #91 shared 95.6 % pairwise identity with WaaL of *Klebsiella pneumoniae* subsp. *pneumoniae* NTUH-K2044 (GenBank accession number NC_012731), which had previously been shown to belong to LPS core type 1 (67). WaaL of AF146532 shared 98.9 % pairwise identity with that of NC_012731. The ClustalW 2.1 alignment was created in Geneious Prime v2019.2.1 using the BLOSUM 62 matrix.

(a)

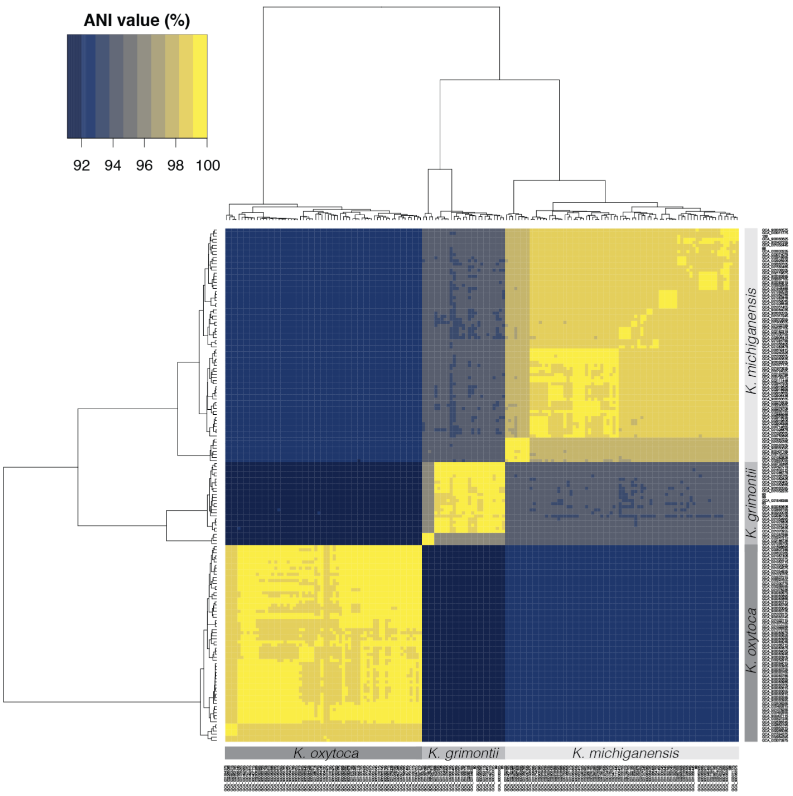
ANI value (%)
92
94
96
98
100
K. michiganensis
K. grimontii
K. oxytoca
K. oxytoca
K. grimontii
K. michiganensis

(b)

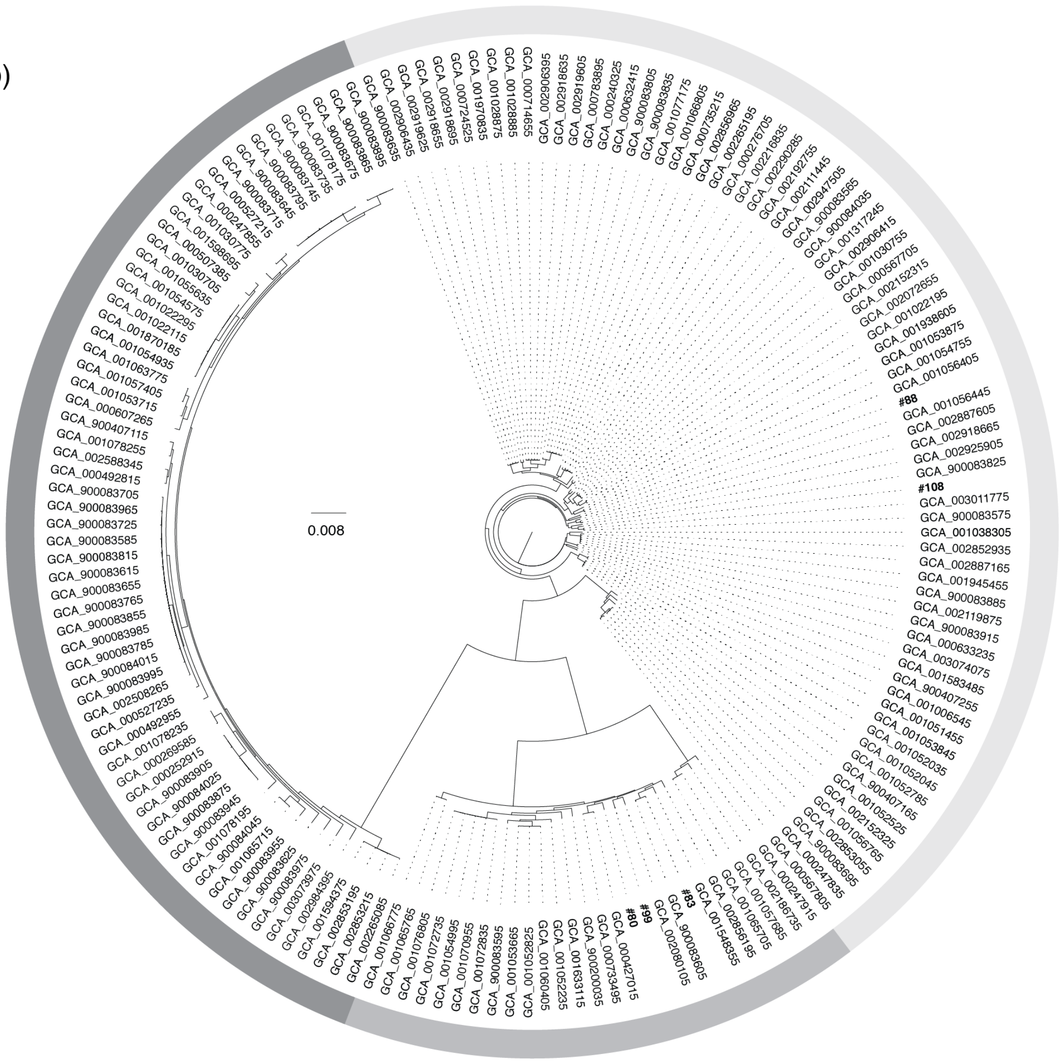
0.008
#88
#108
#80
#99
#83

**Supplementary Figure 5.** Confirmation that the *K. oxytoca*-related isolates recovered from infants were strains of *K. michiganensis* and *K. grimontii*. (a) Heatmap generated with the R package heatmap.2() from FastANI (24) outputs. (b) Phylogenetic tree showing the placement of the genomes of the isolates among confirmed strains of *K. oxytoca*, *K. michiganensis* and *K. grimontii* (36). FastTree v2.1.10 (39) was used to generate the tree from the core gene alignment produced by v3.12.0 (default settings) (38), with the tree visualized using FigTree v1.4.4 (http://tree.bio.ed.ac.uk/software/figtree/). In (a, b), affiliation of strains #88 and #108 with *K. michiganensis* is confirmed, while strains #80, #83 and #89 belong to *K. grimontii*.

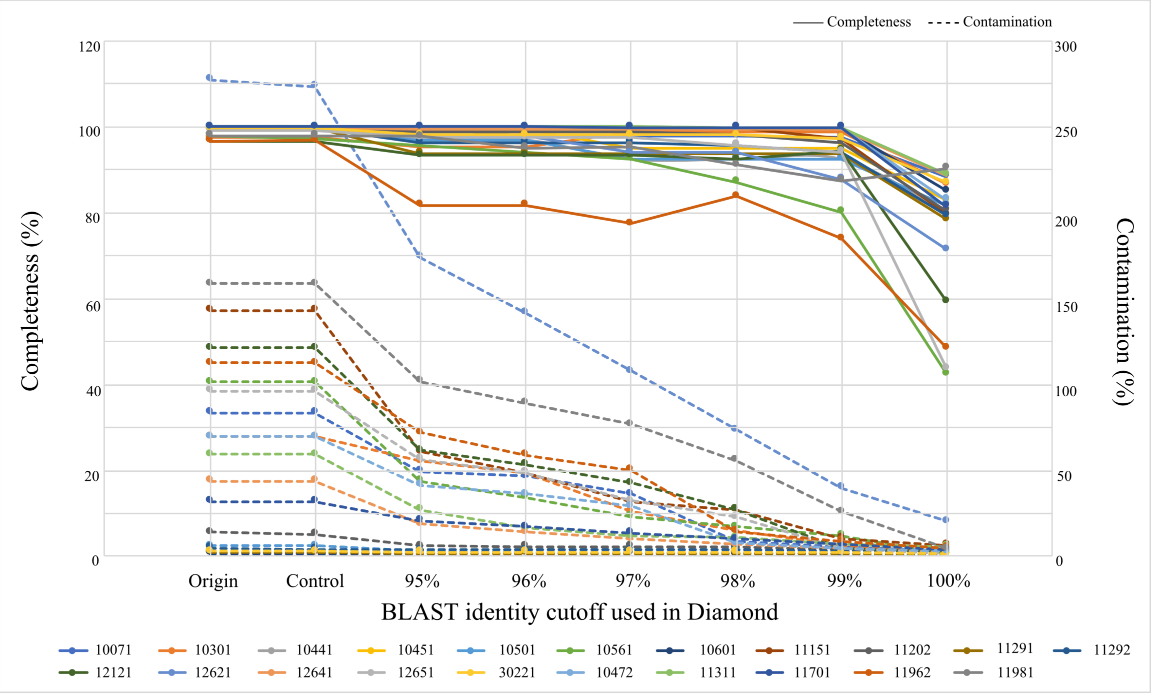


**Supplementary Figure 6.** Genome quality assessment of MAGs. The change of genome completeness and percentage contaminations when different Diamond BLAST identities were applied to MAGs recovered from stage2 samples. Solid line represents completeness and dotted line represents contamination. An identity of 99 % is generally the most suitable cut-off to do decontamination as genome completeness is kept at a reasonable level but the removal of contaminants is effective.

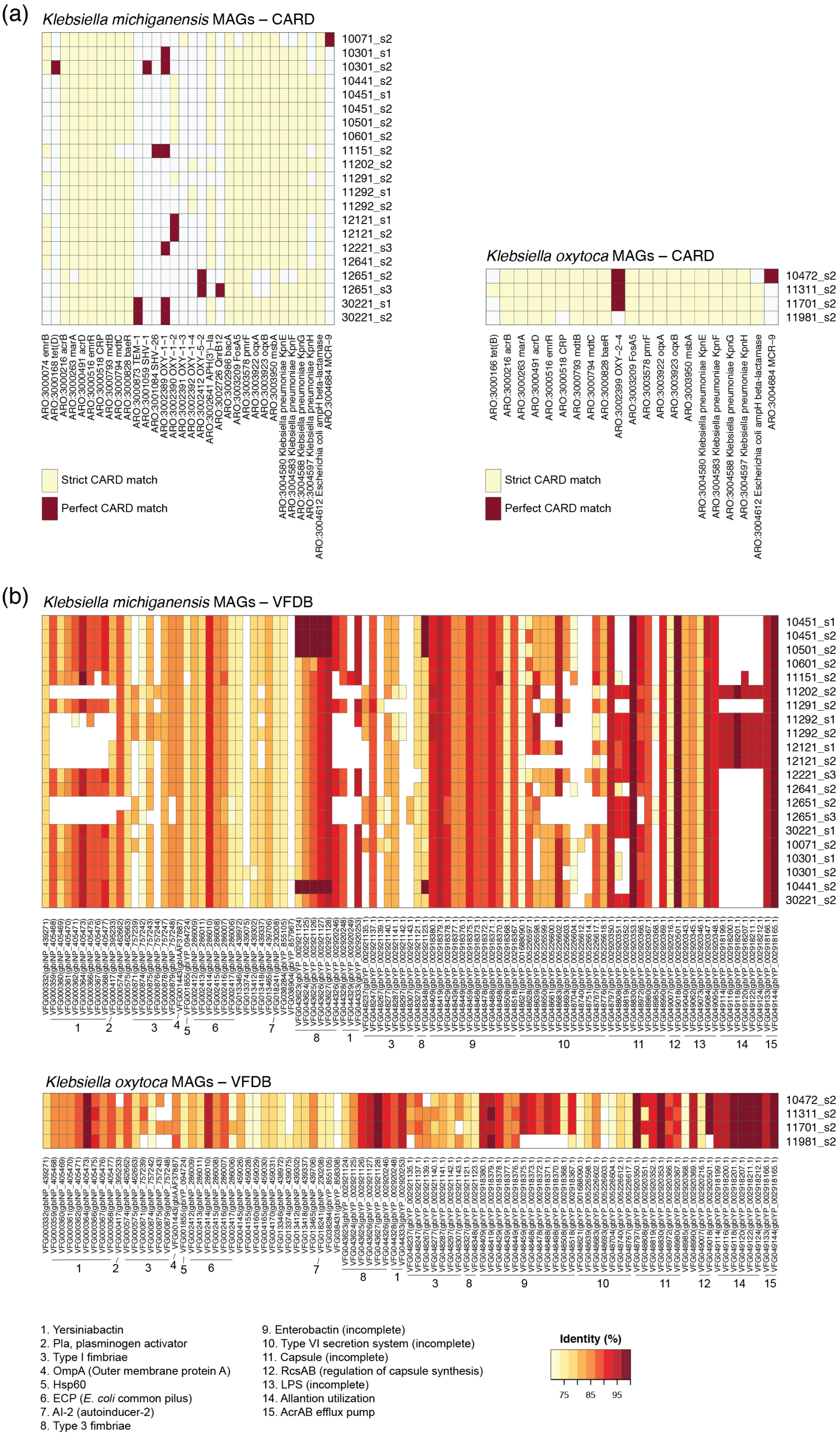
(a)
Klebsiella michiganensis MAGs – CARD
10071_s2
10301_s1
10301_s2
10441_s2
10451_s1
10451_s2
10501_s2
10601_s2
11151_s2
11202_s2
11291_s2
11292_s1
11292_s2
12121_s1
12121_s2
12221_s3
12641_s2
12651_s2
12651_s3
30221_s1
30221_s2
ARO:3000074 emrB
ARO:3000168 tet(D)
ARO:3000216 acrB
ARO:3000263 marA
ARO:3000491 acrD
ARO:3000516 emrR
ARO:3000518 CRP
ARO:3000793 mdtB
ARO:3000794 mdtC
ARO:3000828 baeR
ARO:3000873 TEM–1
ARO:3001059 SHV–1
ARO:3001084 SHV–26
ARO:3002389 OXY–1–2
ARO:3002390 OXY–1–1
ARO:3002391 OXY–1–3
ARO:3002392 OXY–1–4
ARO:3002412 OXY–5–2
ARO:3002641 APH(3')–Ia
ARO:3002726 QnrB12
ARO:3002986 bacA
ARO:3003209 FosA5
ARO:3003578 pmrF
ARO:3003922 oqxA
ARO:3003923 oqxB
ARO:3003950 msbA
ARO:3004580 Klebsiella pneumoniae KpnE
ARO:3004583 Klebsiella pneumoniae KpnF
ARO:3004588 Klebsiella pneumoniae KpnG
ARO:3004597 Klebsiella pneumoniae KpnH
ARO:3004612 Escherichia coli ampH beta-lactamase
ARO:3004684 MCR–9
Strict CARD match
Perfect CARD match
Klebsiella oxytoca MAGs – CARD
10472_s2
11311_s2
11701_s2
11981_s2
ARO:3000166 tet(B)
ARO:3000216 acrB
ARO:3000263 marA
ARO:3000491 acrD
ARO:3000516 emrR
ARO:3000518 CRP
ARO:3000793 mdtB
ARO:3000794 mdtC
ARO:3000828 baeR
ARO:3002399 OXY–2–4
ARO:3003209 FosA5
ARO:3003578 pmrF
ARO:3003922 oqxA
ARO:3003923 oqxB
ARO:3003950 msbA
ARO:3004580 Klebsiella pneumoniae KpnE
ARO:3004583 Klebsiella pneumoniae KpnF
ARO:3004588 Klebsiella pneumoniae KpnG
ARO:3004597 Klebsiella pneumoniae KpnH
ARO:3004612 Escherichia coli ampH beta-lactamase
ARO:3004684 MCR–9
Strict CARD match
Perfect CARD match
(b)
Klebsiella michiganensis MAGs – VFDB
10451_s1
10451_s2
10501_s2
10601_s2
11151_s2
11202_s2
11291_s2
11292_s1
11292_s2
12121_s1
12121_s2
12221_s3
12641_s2
12651_s2
12651_s3
30221_s1
10071_s2
10301_s1
10301_s2
10441_s2
30221_s2
1 2 3 4 5 6 7 8 1 3 8 9 10 11 12 13 14 15
Klebsiella oxytoca MAGs – VFDB
10472_s2
11311_s2
11701_s2
11981_s2
1 2 3 4 5 6 7 8 1 3 8 9 10 11 12 14 15
1. Yersiniabactin
2. Pla, plasminogen activator
3. Type I fimbriae
4. OmpA (Outer membrane protein A)
5. Hsp60
6. ECP (E. coli common pilus)
7. AI-2 (autoinducer-2)
8. Type 3 fimbriae
9. Enterobactin (incomplete)
10. Type VI secretion system (incomplete)
11. Capsule (incomplete)
12. RcsAB (regulation of capsule synthesis)
13. LPS (incomplete)
14. Allantion utilization
15. AcrAB efflux pump
Identity (%)
75
85
95

**Supplementary Figure 7.** Summary of (a) antibiotic resistance and (b) virulence factor genes found in the MAGs recovered from the data of Ward *et al.* (1) by comparison of protein sequences with those of the CARD and VFDB, respectively. (a) Strict CARD match, not identical but the bit-score of the matched sequence is greater than the curated BLASTP bit-score cut-off; perfect CARD match, 100 % identical to the reference sequence along its entire length. Loose matches are not shown to avoid presenting false positives based on sequences with low homology and bit-scores below CARD BLASTP cut-off recommendations. (b) Identity (%), BLASTP reported only for those proteins sharing >70 % identity and 90 % query coverage with VFDB protein sequences.
